# Inducing a chiral twist in achiral porphyrins: tailoring molecular interactions for circularly polarized luminescence from chiral microflowers

**DOI:** 10.1039/d5sc09327a

**Published:** 2026-05-08

**Authors:** Arunima Cheran, Betsy Marydasan, Saikat Das, Hrishikesan Kalpakassery Pattam, Jatish Kumar

**Affiliations:** a Department of Chemistry, Indian Institute of Science Education and Research (IISER) Tirupati Tirupati Andhra Pradesh 517619 India jatish@iisertirupati.ac.in; b Department of Chemistry, Government Arts College Thiruvananthapuram Kerala 695014 India

## Abstract

Circularly polarized luminescent (CPL) materials are gaining prominence in next-generation optoelectronic technologies, including 3D displays, optical data storage, smart sensors and chiroptical light sources for asymmetric synthesis. The inherent limitations associated with tedious synthetic protocols has limited the use of a wide variety of organic systems as CPL emitters. In this work, we report a novel and facile strategy to achieve chiral light emission from achiral porphyrin supramolecular assemblies, thereby overcoming the inherent challenges associated with the use of this class of molecules in CPL investigations. A series of porphyrin derivatives appended with four dipicolylamine units, in the presence of l/d-mandelic acid and zinc(ii) ions, formed supramolecular aggregates exhibiting chiroptical responses. The porphyrin undergoes self-assembly following an isodesmic model of polymerization, resulting in uniform chiral microflowers exhibiting high CPL activity. Systematic investigations on the specific interactions at the molecular level helped probe the mechanism of ground and excited-state optical activity. Notably, when incorporated in polymeric films, the self-assembled structures retained their CPL activity, showcasing the potential application of the microflowers as chiral light emitting materials. The fundamental molecular understanding of chiral induction in achiral porphyrin derivatives paves the way for the development of CPL-active materials across a broader range of organic fluorophores.

## Abstract 2

A facile and novel strategy for induction of chirality in achiral porphyrins through the synergistic use of chiral mandelic acid and Zn(ii) ions is reported. The nanostructures exhibit chiral luminescence in both solution and solid states.

## Graphical abstract

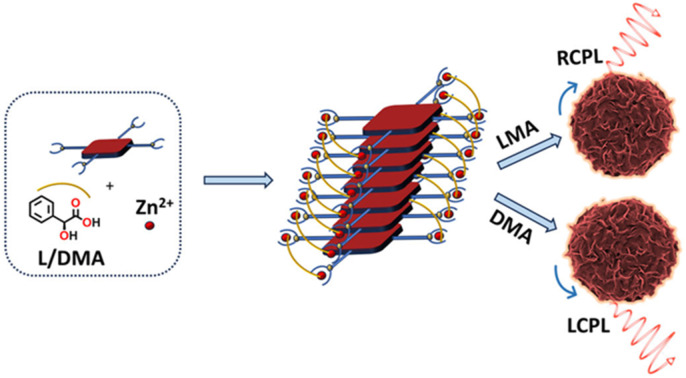

## Introduction

Circularly polarized luminescence (CPL), which analyses the differential emission of left and right circularly polarized light, has emerged as an efficient tool for probing the excited state chiroptical properties.^1,2^ In light of its promising applications in sensing, asymmetric synthesis, display technologies, and optical data storage, the development of CPL-active materials has witnessed a significant surge in recent years.^3,4^ However, achieving high dissymmetry values (*g*_lum_) in simple organic systems is challenging. The luminescence dissymmetry (*g*_lum_) can be theoretically expressed as *g*_lum_ = 4|*m*|cos *θ*/|*µ*|, where *m* and *µ* are magnetic and electric transition dipole moments, respectively, and *θ* is the angle between them.^5^ The inherently weak magnetic dipole contribution in organic systems renders them weak CPL emitters. Efforts to overcome this intrinsic limitation have been through the adoption of various strategies of chiral induction. Chirality can be introduced into luminescent molecules through various approaches, including (i) covalent attachment of stereogenic centers and intrinsically chiral scaffolds (*e.g.*, helicenes or binaphthyls),^6–11^ (ii) supramolecular assembly *via* noncovalent interactions with chiral additives,^12,13^ and (iii) host–guest complexation with chiral templates.^14–17^ Along with the stated approaches, controlled self-assembly serves as a powerful tool for achieving amplified CPL activity, allowing the translation of molecular chirality into highly ordered, hierarchical architectures.^18–22^ By facilitating long-range chiral ordering and excitonic coupling, self-assembly can significantly amplify the chiral dissymmetry of emitted light, yielding robust CPL signals and opening new opportunities for the design of advanced chiroptical systems.^23^

Porphyrins are well-known heterocyclic macrocycles that have captured significant attraction in the field of supramolecular chemistry due to their unique π-conjugated structure, aggregation ability and fascinating optical properties.^24–26^ Owing to their distinctive characteristics, such as planar geometry, metal-coordination sites, and versatile peripheral functionalization, porphyrins are widely recognized for their ability to assemble into higher-order supramolecular structures.^27–33^ Hence, supramolecular assemblies of porphyrins are considered ideal candidates for CPL active materials. However, research in this direction has been scarce, primarily due to their highly symmetric, planar macrocycle and extensive π-conjugation, resulting in dominant electric dipole transitions and weak magnetic dipole contributions. Covalent functionalization of porphyrins with chiral groups often involves tedious, multi-step synthesis with low overall yields.^34–39^ Another approach involving host–guest interactions with chiral templates often relies on complex hierarchical assemblies, making it challenging to decipher the stepwise mechanism of chirality transfer and expression. In contrast, an alternative and highly effective strategy is to utilize chiral small molecules to impart optical activity to achiral porphyrins. This approach allows for precise and well-defined binding events that can be systematically varied and quantified, providing a robust platform for probing the molecular forces, including hydrogen bonding, metal–ligand coordination, and ion pairing, that govern the induction and propagation of chirality. This approach provides a unique opportunity to unravel the mechanistic origins of chiroptical activity in porphyrin assemblies, paving the way for the rational design of CPL-active materials that avoids the need for complex synthetic modifications.^40^ There have been a few reports on the supramolecular assembly of porphyrins using counterions.^41–43^ However, chirality could be achieved only in the ground state with low anisotropy values. Achieving excited state optical activity in achiral porphyrins through chiral additives has been a challenge, and hence, remains largely unexplored. This work presents a simple yet powerful strategy for generating strong CPL from inherently achiral porphyrins through supramolecular self-assembly, offering a practical and broadly applicable route to chiral light-emitting materials without demanding tedious synthetic modification. We herein design a facile approach to induce chirality in achiral porphyrins using a chiral additive and metal ions. A tetrasubstituted dipicolylamine porphyrin in the presence of l/d-mandelic acid (l/d-MA) and Zn(ii) ions resulted in ground and excited state chiroptical responses. The peculiar structural features of the molecule provide specific binding sites for the chiral molecule and the metal ion, leading to enhanced optical activity. The mechanism as well as the nature of interaction during self-assembly was probed comprehensively using various spectroscopic and microscopic techniques. Understanding and harnessing the amplification mechanism is crucial for the rational design of supramolecular systems with tailored chiral responses.

## Results and discussion

**Fig. 1:**
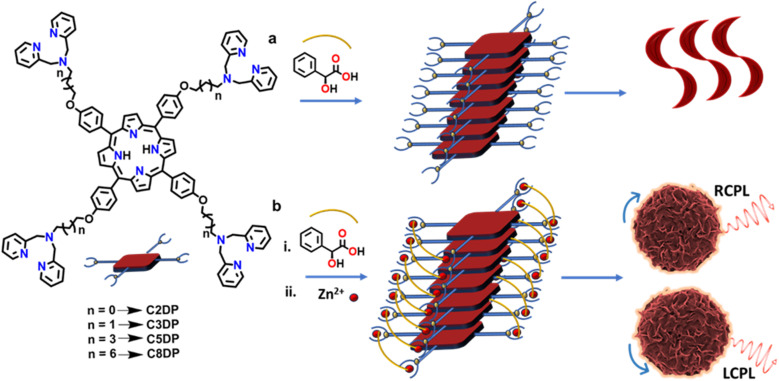
Schematic illustration of the self-assembly of MA–porphyrin in the (a) absence and (b) presence of zinc ions. Molecular structures of the synthesized dipicolylamine substituted porphyrin derivatives are provided.

**Fig. 2:**
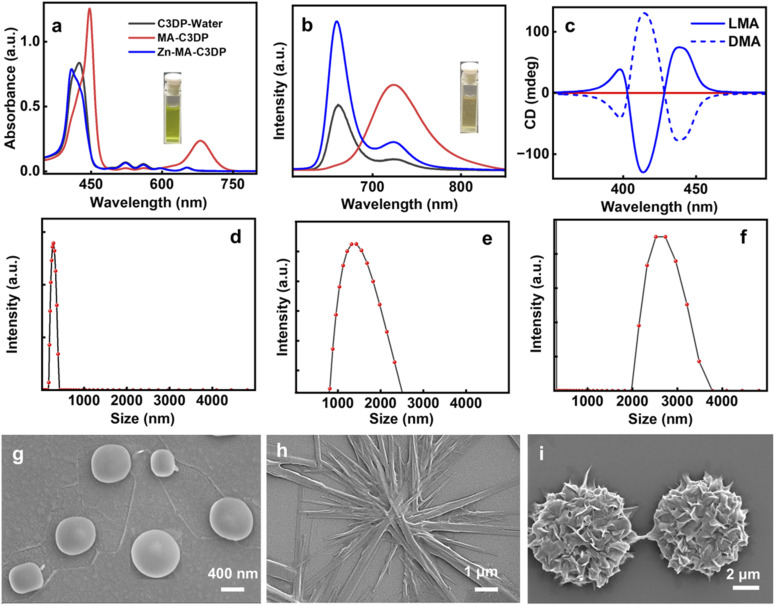
(a) UV-visible absorption and (b) fluorescence spectra of C3DP in a mixture of DMSO : water (1 : 99) (black trace), C3DP-MA (red trace) and C3DP-MA-Zn (blue trace). (c) CD spectra of C3DP-MA (red traces) and C3DP-MA-Zn (blue traces) in the presence of l-MA (solid line) and d-MA (dotted line). DLS spectra and SEM images of (d and g) C3DP, (e and h) C3DP-MA and (f and i) C3DP-MA-Zn, respectively, in a DMSO : water (1 : 99) mixture.

A series of tetrasubstituted dipicolylamine porphyrins with varying alkyl chain lengths (C2DP, C3DP, C3P, C5DP and C8DP) were synthesized adopting a multistep procedure (Fig. 1 and S1). Alkylation of 4-hydroxy benzaldehyde was achieved using bromoalkane and K_2_CO_3_. Subsequently, the meso-substituted symmetrical porphyrin was synthesized adopting a Rothemund–Lindsey method *via* monopyrrole tetramerization. The bromo groups in the alkyl chain were substituted with dipicolylamine units in the presence of KI and K_2_CO_3_. The synthesized compounds were characterized using NMR, HRMS and MALDI-TOF, which supported their structural identities (Fig. S2–S34). Owing to the high polarity of the compounds and their propensity for aggregation, the NMR signals appeared slightly broadened with reduced intensity under the measurement conditions. The photophysical investigations of C3DP in DMSO revealed a sharp Soret band at 424 nm and less intense Q bands at 519, 556, 598 and 653 nm (Fig. S35a), features typical of porphyrins. The molecule upon excitation at 424 nm exhibited emission spectra at 660 nm with a shoulder peak at 723 nm (Fig. S35b). The excited state fluorescence lifetime measurements showed a monoexponential fit with a value of 9.56 ns (Fig. S36a). The molecule underwent aggregation in a DMSO : H_2_O (1 : 99) mixture (Fig. S37), wherein the UV-vis and fluorescence spectra exhibited a hypochromic shift and broadening (black trace in Fig. 2a and b). Upon aggregation, the fluorescence lifetime exhibited a biexponential decay with *τ*_1_ = 1.13 ns (24.57%) and *τ*_2_ = 9.28 ns (74.43%) (Fig. S36b). The aggregated structures exhibited a spherical morphology with an average size of 700 nm (Fig. 2g). However, the molecules in their monomeric and aggregated states were found to be optically inactive (Fig. S35c and d).

In order to induce chirality into the molecular systems, l/d-MA was chosen as the chiral additive.^44^ MA is a chiral α-hydroxy acid that is capable of inducing and directing a chiral bias within supramolecular porphyrin assemblies.^45–48^ MA can bind to metalloporphyrins *via* coordination or H-bonding. MA addition to C3DP porphyrin in water led to remarkable spectral changes: the Soret band at 424 nm exhibited a significant bathochromic shift to 450 nm, accompanied by the disappearance of the four Q bands and the formation of a new peak at 680 nm (red trace, Fig. 2a). The fluorescence spectra exhibited a bathochromic shift of 63 nm with the disappearance of the shoulder peak (red trace, Fig. 2b). The UV-vis and fluorescence spectral features are characteristic of the conformational changes associated with the protonation of the porphyrin core.^44,48^ The protonation of the core was further evident from the visual colour change of the solution from faint yellow to intense green (inset, Fig. 2a). Dynamic Light Scattering (DLS) measurements were conducted at each stage of the self-assembly process to evaluate changes in aggregate size. The initial spherical aggregates displayed an average diameter of approximately 700 nm, which increased to 1.5 µm for C3DP-MA and further to 2.5 µm for C3DP-MA-Zn (Fig. 2d–f). The MA protonated porphyrin (C3DP-MA) exhibited a branched fibrillar morphology (Fig. 2h), and similar structures were obtained with both the isomers (Fig. S38a). Contrary to our expectations, the aggregated structures remained optically inactive even in the presence of chiral acid (red trace, Fig. 2c).

Zinc porphyrins are well known to form aggregates due to their planar, π-conjugated macrocyclic structure, which enables strong π–π stacking interactions, and metal–ligand coordination capabilities.^49^ Moreover, the dipicolylamine units are well known for their strong affinity to zinc ions; hence, zinc acetate (Zn(OAc)_2_) was added to the solution to further enhance the aggregation. Upon addition of Zn(ii) ions, the UV-vis spectra exhibited a hypsochromic shift of the Soret band and the reappearance of the four Q bands (blue trace, Fig. 2a). Fluorescence spectra also exhibited a hypsochromic shift of 64 nm, accompanied by the regaining of the shoulder band at 724 nm (blue trace, Fig. 2b). The intense green colour of the solution turned back to faint yellow on zinc addition (inset, Fig. 2b). The spectral features and the visual appearance are indicative of the porphyrin retaining its *D*_2h_ symmetry, wherein the core is free of metal coordination.^50,51^ The ultimate goal of the study being the chiroptical investigation, CD spectra of the solution were collected. Interestingly, intense bisignate CD signals in the Soret region with a zero crossover at 403 and 428 nm were obtained from solutions in the presence of zinc, suggesting the excitonic coupling between porphyrin chromophoric units arranged in a non-planar geometry. The mirror image CD profile for solutions containing l- and d-MA confirmed the role of the chiral acid in inducing optical activity in the porphyrin nanostructures (Fig. 2c). The porphyrins exhibited a chiral anisotropy in absorption (*g*_abs_) of +3.0 × 10^−3^ and −3.2 × 10^−3^ for solutions in the presence of l- and d-MA, respectively. Interestingly, morphological characterization revealed the presence of uniform flower-like structures throughout the sample (Fig. 2i). Similar structures formed with the opposite isomer of MA confirmed the morphological characteristics (Fig. S39). Hence, the addition of zinc clearly indicates a structural transformation of the aggregates from a fibrillar morphology to flower-like geometry. The absolute quantum yield was measured at each stage of the process. C3DP in DMSO exhibited a quantum yield of 26.9%, while in DMSO : water (1 : 99) the value reduced to 5%. The quantum yield of C3DP-MA was found to be 10%, which increased to 15% in C3DP-MA-Zn. The excited-state lifetime measurements of C3DP-MA and C3DP-MA-Zn exhibited a biexponential fit, with the former exhibiting lifetimes of *τ*_1_ = 2.18 ns (90.74%) and *τ*_2_ = 10.38 ns (9.26%), whereas the latter showed values of *τ*_1_ = 2.6 ns (25.31%) and *τ*_2_ = 9.72 ns (74.69%) (Fig. S36c and d).

**Fig. 3:**
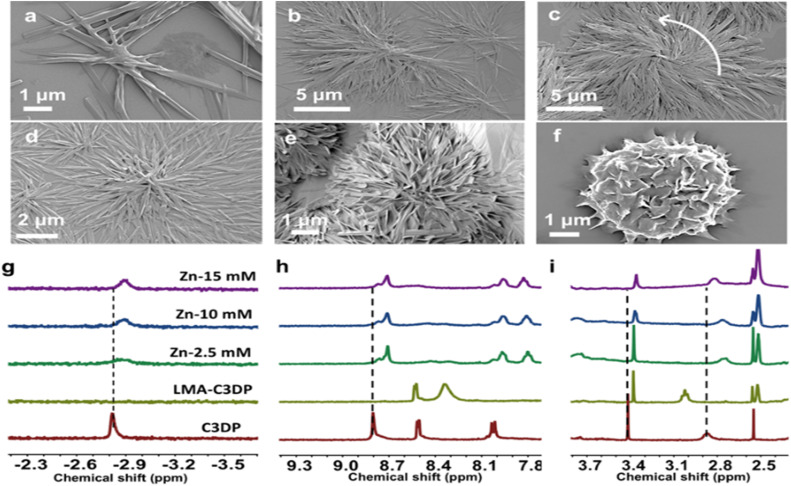
SEM images of C3DP-LMA-Zn assemblies with increasing concentration of Zn(ii): (a) 0 mM, (b) 2.5 mM, (c) 5 mM, (d) 7 mM, (e) 12.5 mM and (f) 14.5 mM. (g–i) ^1^H NMR titration of C3DP in DMSO-d_6_ with stepwise and sequential addition of MA in D_2_O followed by Zn(OAc)_2_ in D_2_O.

In order to investigate the mechanism of self-assembly of achiral molecules into chiral aggregates, a thorough investigation was carried out by systematically varying the MA and zinc ratio. Upon gradual addition of zinc ions to the protonated solution of C3DP (0.01 mM), significant changes in the morphology were observed due to the interactions between the dipicolylamine units and the zinc ions. By increasing the concentration of zinc from 0 to 2.5 mM at a fixed MA concentration of 2.5 mM, the extent of self-assembly was enhanced significantly, leading to the formation of larger aggregates (Fig. 3b); however, the structures were found to be CD silent at this stage (Fig. S40). Upon increasing the concentration to 5 mM, a noticeable anticlockwise chiral twist was evident (Fig. 3c and d). With further addition of zinc (7 mM), the twisted structures underwent entangling, leading to the formation of a well-defined flower-like morphology (Fig. 3e). CD signals emerged at this point, although with relatively low intensity. On further addition of zinc (14.5 mM), a well-defined flower-like morphology was observed (Fig. 3f & S39). These flower-like structures consisted of twisted nanolayers and exhibited intense CD signals (Fig. S40c). Similar structures were observed for the opposite isomer with varying concentrations of zinc, but with a clockwise chiral twist (Fig. S38c). The binding constant for the interaction between porphyrin and zinc(ii) was determined using Benesi–Hildebrand analysis by employing the UV-vis titration data (Fig. S41). The binding constant obtained through this calculation was found to be 11.36 × 10^2^ M^−1^. An excess of mandelic acid and zinc (∼100 eq.) was employed to achieve chirality induction in aqueous solution, owing to the strong competition between water molecules and mandelic acid for hydrogen-bonding interactions with the porphyrin core.

To understand the interactions operative during the self-assembly, ^1^H NMR titration experiments were carried out. During the ^1^H NMR titration, only minimal D_2_O was added to prevent excessive aqueous content and unwanted aggregation. Concentrated stock solutions of MA and zinc acetate were used to maintain uniform conditions and minimize dilution effects.

When MA in D_2_O was gradually added to C3DP dissolved in DMSO-d_6_, the β-pyrrole proton signal at 8.8 ppm showed a noticeable upfield shift, indicating π–π stacking interactions operating during the aggregation (Fig. 3h & S42a).^52^ This shift is attributed to a distortion from planarity, which alters the porphyrin's diamagnetic anisotropy and reduces its ring current. A closer look at the aromatic region showed no evidence of protonation of the pyridine rings in the dipicolylamine (DPA) core. Typically, protonated pyridine (pyridinium) groups act as strong electron-withdrawing units, causing downfield shifts in both their own protons and nearby β-pyrrole protons. However, since the aromatic protons of the DPA units did not shift downfield, we conclude that protonation did not occur at these sites (Fig. S42e).^53^ Protonation of the inner nitrogen atoms of the porphyrin core can distort the macrocycle, rendering proton exchange with the solvent easier and faster. This explains the disappearance of the inner N–H signal at −2.8 ppm (Fig. 3g & S42b).^52^ Additionally, aliphatic protons at 3.9 and 2.8 ppm showed deshielding, likely due to core protonation (Fig. 3i, S42c and d). In contrast, a shielding of the aliphatic protons at 3.4 ppm suggested π–π stacking between DPA units (Fig. S42d).^54^ Upon the addition of zinc, a highly shielded peak in the negative region reappeared (Fig. 3g), and the β-pyrrole and aliphatic protons appeared more deshielded compared to the protonated porphyrin (Fig. 3h and i). This indicates that zinc coordinates with the DPA nitrogen, and the porphyrin core restores the symmetry. From the obtained results, we propose that when the porphyrin core is protonated, electron donation from the DPA unit to the core takes place. This suppresses the formation of a strong ion pair between porphyrin and MA. However, upon zinc addition, the DPA units get coordinated to Zn(ii), and the charge transfer is blocked.^55,56^ Zinc can then interact with the MA anion, enabling effective chirality induction in the porphyrin assembly leading to intense chiroptical signals.

**Fig. 4:**
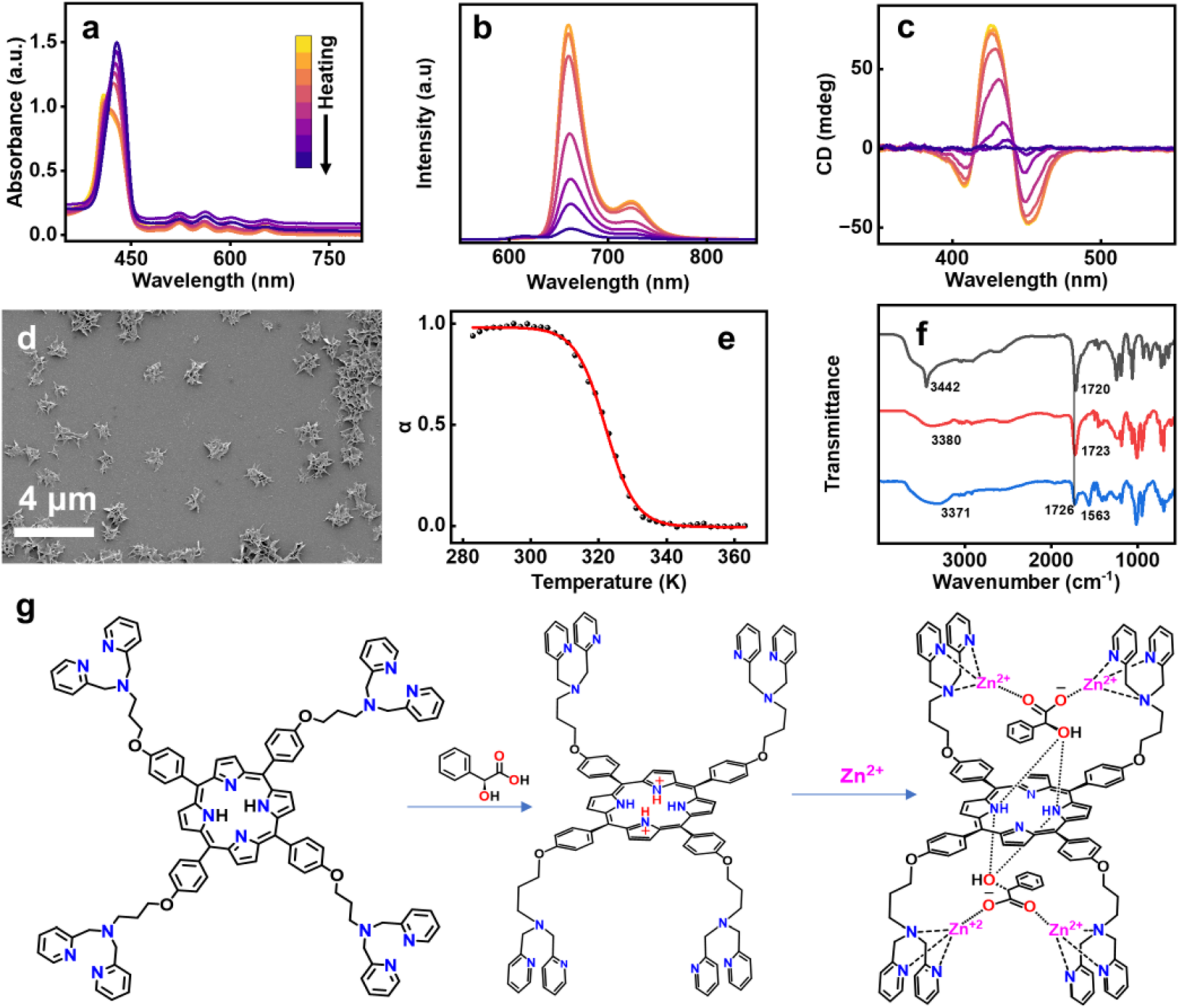
Temperature dependent (a) UV-vis, (b) fluorescence and (c) CD spectra of C3DP-MA-Zn aggregates in DMSO : water (1 : 99). (d) SEM image of the aggregates after the heating–cooling cycle. (e) Plot of the degree of aggregation *versus* temperature. (f) FTIR spectra of C3DP upon gradual addition of MA and zinc (black trace – MA in water, red trace – C3DP-MA and blue trace – C3DP-MA-Zn). (g) Scheme illustrating the noncovalent interactions driving the chiral self-assembly of the porphyrins.

To obtain a comprehensive understanding of the mechanism of supramolecular polymerization, variable temperature UV-vis absorption, fluorescence, and CD spectra were recorded. Upon heating the solution from 5 °C to 95 °C at an interval of 10 °C min^−1^, the UV-vis spectra underwent a bathochromic shift. This was accompanied by a quenching of fluorescence that remained irreversible even on cooling back to room temperature (Fig. 4a, b, S43a and b). Moreover, heating led to complete disappearance of CD, which showed no reversal on cooling (Fig. 4c & S43c). SEM images display the disassembly of the flower-like ordered arrangement of the chiral aggregates (Fig. 4d). CD spectral changes as a function of temperature was employed as an effective tool to analyze the mechanism of polymerization. A plot of temperature against the degree of aggregation resulted in a sigmoidal curve, indicating that the assembly proceeded *via* an isodesmic model of polymerization (Fig. 4e). The temperature dependent sigmoidal curve was fitted using the process described by van der Schoot.^57^ Fitting these values to eqn (1) provides the value of association constant (K), melting temperature (*T*_m_), and changes in enthalpy (Δ*H*) and entropy (Δ*S*).1
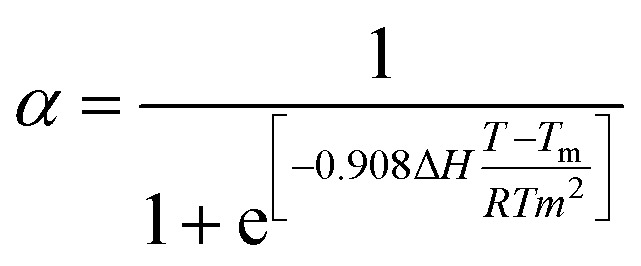


The Δ*H* was calculated to be −216 kJ mol^−1^ with a melting temperature of 322 K. The Δ*S* calculated from Δ*H* was found to be −588.67 J mol^−1^. A favourable Gibbs free energy of −41.47 kJ mol^−1^ aided the reaction in the forward direction. The binding constant was calculated to be 7.77 × 10^6^ M^−1^, a relatively high value in the realm of supramolecular polymerization. To validate these results, temperature-dependent CD measurements were performed at three additional concentrations (Fig. S44), and the corresponding thermodynamic parameters were determined. All calculated values fell within a consistent range, confirming the reproducibility of the measurements (Table S1). In order to probe the mechanism further, FT-IR spectra were collected for MA, C3DP-MA and the C3DP-MA-Zn assembly. A sharp peak at 3442 cm^−1^ corresponding to the O–H stretch was observed for MA, which shifted to 3380 cm^−1^ on interacting with the porphyrin, depicting the hydrogen bonding interactions operating between porphyrin and MA.

In C3DP-MA-Zn assembly, a broad O–H stretching frequency at 3371 cm^−1^ was observed, which proves the enhanced hydrogen bonding interactions on zinc addition. The strength of the C=O bond is affected by the presence of metal ions between C=O and the picolyl nitrogen, causing a proton–cation exchange at carboxylic acid resulting in carboxylate groups (Fig. 4g).^58^ This interaction causes a decrease in the intensity of the COOH band, accompanied by a shift from 1730 to 1710 cm^−1^, while a COO^−^ band appears in the range of 1620–1550 cm^−1^ (Fig. 4f). The frequency of the asymmetric carboxylic vibration in the FTIR spectra and the magnitude of separation between the carboxylate stretches are often used to determine the mode of carboxylic acid binding.^59^ The separation between the two asymmetric stretching (1726 & 1563 cm^−1^) was found to be in agreement with the monodentate binding between the two Zn(ii) ions coordinated to the dipicolylamine units and one counter ion of MA (Fig. 4g). Previous studies on the mandelate complex of *D*_2_-symmetric saddle-shaped porphyrins have reported the formation of monodentate hydrogen-bonding to the inner pyrrolic NH moieties of porphyrin.^45^ Despite the well-defined binding mode, the resulting complex exhibited dynamic behaviour, leading to rapid ring inversion and loss of chiral integrity. In contrast, the choice of dipicolylamine units in C3DP facilitates effective coordination with Zn(ii), promoting the formation of a tightly bound ion pair with mandelic acid.^55,56^ The judicious choice of molecular functionalities aids effective interactions that suppress ring inversion and preserve the induced chirality. Consequently, the chirality of the mandelic acid counterion is effectively transferred to the porphyrin macrocycle. This chirality transfer promotes the formation of twisted chiral structures that act as fundamental building blocks for the formation of the well-defined flower-like morphology.

With an objective of investigating the effect of variation in the length of the alkyl chain linker on the nature of aggregation, the photophysical and chiroptical properties of C2DP, C5DP and C8DP derivatives were studied. C2DP exhibited spectral features similar to C3DP with a slightly lower g_abs_ value of +2.5 × 10^−3^ and −2.2 × 10^−3^ for l- and d-MA, respectively (Fig. S45c). Analogous to studies on C3DP, C2DP exhibited flower-like morphology in the presence of MA and zinc (Fig. S45d–f). In contrast, C5DP and C8DP displayed no CD signals under similar experimental conditions, emphasizing the relevance of the chain length in aggregation and thereby the chiral induction (Fig. S46c & S47c). In contrast to C2DP and C3DP, which exhibited well-defined flower-like morphologies, C5DP and C8DP failed to form such defined hierarchical structures, thereby rationalizing the absence of chirality induction in these systems (Fig. S46d–f & S47d–f). Based on these observations, it could be concluded that an optimal distance between the porphyrin core and dipicolylamine units is of relevance for the favourable interaction with the MA counterion. The mechanism of supramolecular polymerization was studied in the case of C2DP, and the thermodynamic parameters were comparable to those obtained for C3DP (Fig. S48).

UV-visible absorption, fluorescence and CD spectra of C3DP-MA-Zn aggregates (0.01 mM) were collected with varying concentrations of MA and zinc. The intensity of CD spectra increased as the concentration of zinc was increased from 2.5 mM to 25 mM, while maintaining a constant MA concentration of 2.5 mM. Further addition of zinc had no effect on the CD signal, concluding that there is no further growth of supramolecular structures (Fig. S40). Similarly, on increasing the concentration of MA from 1 mM to 12 mM, while maintaining a constant zinc concentration of 6.25 mM, a gradual increase in the CD intensity was observed which saturated on further addition of MA (Fig. S49). Similar observation was obtained for the C2 derivative as well (Fig. S50 and S51). In order to study the specific role of MA in chirality induction, other chiral acids such as camphor sulphonic acid (CSA) and polyglutamic acid (PGA) were used (Fig. S52). Weak chirality observed in the presence of these acids reveals the vital role of hydrogen bonding interactions between the counterion of MA and porphyrins in chirality induction. Similarly, the ability of other metal ions in chirality induction was investigated by titrating the protonated form of C3DP in water, with a small amount of metal salts dissolved in water. Metal ions such as Cu^2+^, Cd^2+^, Hg^2+^, Eu^3+^, and Tb^3+^ were selected for this purpose. No chirality induction could be observed for any metals other than zinc (Fig. S53). Hence, the affinity of dipicolylamine to Zn(ii) is vital to the formation of metal coordination bonds, which lead to chirality induction. As a control study, four-sided picolylamine substituted porphyrin (Fig. S18 and S19) was synthesized, and its chiroptical properties were investigated (Fig. S54). The molecule failed to show any chiroptical activity, which emphasizes the role of dipicolylamine units in binding to the Zn(ii) ions and providing a favourable orientation for chirality induction. The order of addition in porphyrin supramolecular assembly determines the assembly pathway, morphology, and chiral expression. Hence, the sequence of addition of reagents was varied, and the spectral changes were recorded. Upon addition of zinc to the porphyrin assembly in water followed by l-/d-MA, no significant changes were observed in the absorption or fluorescence spectra. This behaviour contrasts with the reverse order of addition, where distinct spectral changes occurred at each step, indicating a difference in the mode of interaction or assembly pathway (Fig. S55a and b). The CD signals were also found to be extremely weak at this stage (Fig. S55c). To further clarify this behavior, a reverse ^1^H NMR titration was carried out (Fig. S56). Upon addition of Zn(ii) to C3DP in DMSO-d_6_, the inner pyrrole protons remained visible, indicating that zinc does not coordinate to the porphyrin core under these conditions. In contrast, the subsequent addition of MA resulted in the immediate disappearance of the inner core protons, demonstrating its essential role in enabling coordination. It could be understood that the supramolecular interactions are dynamic and sensitive to conditions. Careful control of the sequence of mixing is essential for reproducible and tunable structures with desired chiroptical responses.

**Fig. 5:**
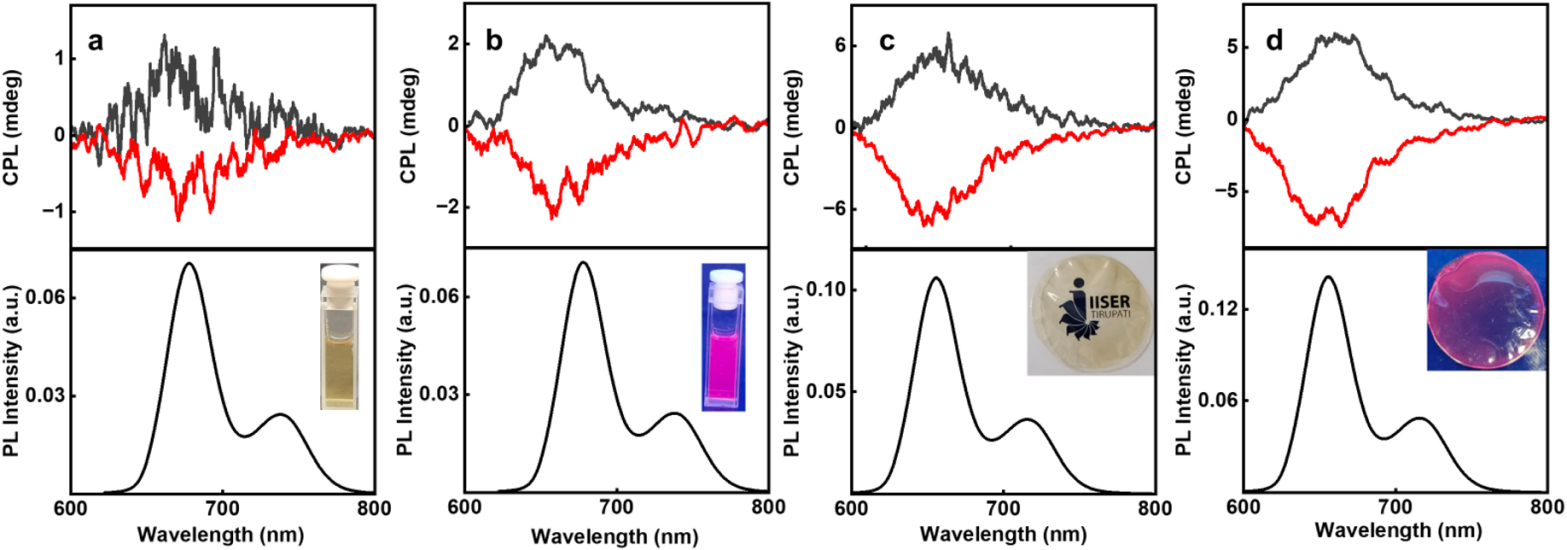
CPL and the corresponding PL spectra of (a and c) C2DP-MA-Zn and (b and d) C3DP-MA-Zn in the (a and b) solution state and (c and d) incorporated in the PVA matrix. Red and black traces represent the spectra of aggregates formed in the presence of d- and l-MA, respectively. Insets show the photographs of the C3DP-MA-Zn solutions and the PVA films containing the self-assembled nanostructures under (a and c) ambient light and (b and d) UV light.

In this case, the initial protonation of the porphyrin core followed by Zn(ii) coordination is vital for chirality induction. The ultimate goal being chiroptical investigations in the excited state, CPL spectra of C2DP and C3DP were collected for samples with l/d-MA and Zn(ii) ions. Interestingly, C2DP-MA-Zn aggregates in the presence of l- and d-MA exhibited positive and negative CPL plots, with a *g*_lum_ of +1.35 × 10^−3^ and −1.38 × 10^−3^, respectively (Fig. 5a). The flower-like structures formed from C3DP-MA-Zn exhibited positive and negative CPL for l- and d-MA, with a *g*_lum_ value of +2.02 × 10^−3^ and −2.1 × 10^−3^, respectively (Fig. 5b). The sign and extent of CPL match well with those of the CD plots, indicating the similar nature of chiral induction in the ground and excited states. Designing solid state samples exhibiting CPL activity is key to finding applications for the optically active nanostructures. With an objective to fabricate solid-state chiral luminescent materials, PVA films incorporating the aggregates were prepared. C2DP-MA-Zn aggregates in PVA films exhibited clear mirror image plots with a *g*_lum_ value of +2.1 × 10^−3^ and −2.3 × 10^−3^, respectively (Fig. 5c). Similarly, C3DP-MA-Zn aggregates exhibited a *g*_lum_ value of +3 × 10^−3^ and −3.01 × 10^−3^ in the presence of l- and d-MA, respectively (Fig. 5d). The magnitude and sign of CPL signals in the solution and solid films were consistent, confirming the retention of optical activity of the aggregated structures even when embedded in the polymer matrix. The fluorescence lifetime of C2 aggregates in the PVA film exhibited a biexponential decay with *τ*_1_ = 3.87 ns (34.01%) and *τ*_2_ = 5.9 ns (65.99%), whereas the C3 aggregates in the PVA film exhibited a biexponential decay with *τ*_1_ = 3.58 ns (23.62%) and *τ*_2_ = 6.2 ns (76.238%) (Fig. S57). Luminescence quantum yields were calculated to be 11 and 16% for the C2DP-MA-Zn and C3DP-MA-Zn films, respectively. CPL was collected from multiple points on the film to confirm the consistency of the signals and to rule out the possibility of any artefacts (Fig. S58 & S59). While the luminescence dissymmetry exhibited may not be extremely high, the values are within the range reported for various organic fluorophores. The novel strategy demonstrated herein paves the way for extending such an approach to a variety of organic systems for the fabrication of similar nanostructures exhibiting enhanced chiral anisotropy.

## Conclusion

In summary, we report a facile and novel strategy for inducing chirality in achiral dipicolylamine-substituted porphyrins through the synergistic use of MA and Zn(ii) ions. Addition of MA to C3DP led to the protonation of the inner core of porphyrins, facilitating a charge transfer from the dipicolylamine unit to the porphyrin core. This interaction, however, is unfavorable for chirality induction from the counter ion of MA, and hence the system remained CD silent. Upon zinc addition, the Zn(ii) ions non-covalently bind to the dipicolylamine handles and block the charge transfer between the peripheral dipicolylamine units and the core protons. Meanwhile, the Zn(ii) ions undergo monodentate binding to the carboxylate group of MA, which provides favorable conditions for chirality induction resulting in a chiral twist. The thermodynamic parameters favoured the formation of optically active aggregated structures. An optimal length of the aliphatic chain is vital to the effectiveness of the interaction, and the resulting chirality induction. Under optimized conditions, π–π stacking interactions, hydrogen bonding and metal coordination led to the formation of a chiral porphyrin assembly exhibiting CD and CPL activity. Comprehensive investigation into the self-assembly process and the nature of molecular interactions revealed the mechanistic basis of the chiral induction. Aggregation resulted in the formation of well-defined flower like morphology that exhibited exciton coupled CD and mirror image CPL in solution. Incorporation of the nanostructures into the PVA film exhibited clear mirror image CPL signals for the two isomers of the nanoaggregates with reasonably high luminescence dissymmetry. This approach offers a versatile platform for the design of CPL-active organic nanosystems, overcoming the conventional complex synthetic routes.

## Author contributions

A. C. and B. M. contributed equally to the work. J. K. conceived and coordinated the project. A. C., B. M., S. D. and H. K. P. carried out the experimental work. A. C., B. M. and J. K. analysed the data and prepared the manuscript. All authors have given approval to the final version of the manuscript.

## Conflicts of interest

There are no conflicts to declare.

## Supplementary Material

SC-OLF-D5SC09327A-s001

## Data Availability

All experimental details are added to the supplementary information (SI) file. Supplementary information: experimental details on the synthesis of molecules, characterization of the synthesized molecules, optical and structural characterization of the chiral nanostructures, and chiroptical properties of the films. See DOI: https://doi.org/10.1039/d5sc09327a.
