# Supplementary material for: Inducing a chiral twist in achiral porphyrins: tailoring molecular interactions for circularly polarized luminescence from chiral microflowers
Source: Chem Sci. 2026 May 8;17(24):11865–73. doi: 10.1039/d5sc09327a (PMC13154874; doi:10.1039/d5sc09327a)
Supplement: SC-017-D5SC09327A-s001 [file SC-017-D5SC09327A-s001.pdf]

Supplementary Information (SI) for Chemical Science.
This journal is © The Royal Society of Chemistry 2026

**Inducing Chiral Twist in Achiral Porphyrins: Tailoring Molecular Interactions for Circularly Polarized Luminescence from Chiral Microflowers**

Arunima Cheran,[a#] Betsy Marydasan,[b#] Saikat Das,[a] Hrishikesan Kalpakassery Pattam,[a] Jatish Kumar[a*]

[a]*Department of Chemistry, Indian Institute of Science Education and Research (IISER) Tirupati, Tirupati 517619, India*

[b]*Department of Chemistry, Government Arts College Thiruvananthapuram, Kerala 695014, India*

Email: jatish@iisertirupati.ac.in


| Sl. No. | Contents | Page no. |
|---|---|---|
| 1 | Experimental section | S4 |
| 2 | Fig. S1. General Synthesis scheme | S5 |
| 3 | Fig. S2 - S34. Characterization data of the compounds | S4 – S28 |
| 4 | Fig. S35. Spectral changes of C3DP in DMSO and water | S29 |
| 5 | Fig. S36. Fluorescence lifetime decay curves at different stages | S30 |
| 6 | Fig. S37. Solvent dependent studies of C3DP | S30 |
| 7 | Fig. S38. SEM images of C3DP-DMA on varying Zn(II) concentration | S31 |
| 8 | Fig. S39. SEM images of C3DP-DMA-Zn | S31 |
| 9 | Fig. S40. Spectral changes of C3DP on varying the Zn(II) concentration | S32 |
| 10 | Fig. S41. Benesi-Hilbrand plot for calculation of binding constant | S32 |
| 11 | Fig. S42. $^1$H NMR titration spectra of C3DP on addition of L/D-MA | S33 |
| 12 | Fig. S43. Temperature dependent spectral changes of C3DP-MA-Zn | S33 |
| 13 | Fig. S44. Temperature dependent spectral changes | S34 |
| 14 | Table S1. Table depicting the thermodynamic parameters | S34 |
| 15 | Fig. S45. Absorption, PL and CD spectra of C2DP-MA-Zn aggregates | S35 |
| 16 | Fig. S46. Absorption, PL and CD spectra of C5DP-MA-Zn aggregates | S35 |
| 17 | Fig. S47. Absorption, PL and CD spectra of C8DP-MA-Zn aggregates | S36 |

| | | |
|---|---|---|
| 18 | Fig. S48. Temperature dependent changes of C2DP-MA-Zn aggregates | S36 |
| 19 | Fig. S49. Spectral changes of C3DP on varying the MA concentration | S37 |
| 20 | Fig. S50. Spectral changes of C2DP on varying the Zn(II) concentration | S37 |
| 21 | Fig. S51. Spectral changes of C2DP on varying the MA concentration | S38 |
| 22 | Fig. S52. Spectral changes of C3DP in the presence of CSA and PGA | S38 |
| 23 | Fig. S53. Spectral changes of C3DP in the presence of various metal ions | S39 |
| 24 | Fig. S54. Spectral changes of C3 picolylamine with MA and Zn(II) | S39 |
| 25 | Fig. S55. Spectral changes of C3DP on varying the sequence of addition | S40 |
| 26 | Fig. S56. $^{1}$H NMR titration spectra of C3DP on varying the order of addition | S40 |
| 27 | Fig. S57. Lifetime plots of samples incorporated into PVA film. | S40 |
| 28 | Fig. S58. CPL spectra of C2 PVA film collected from multiple points | S41 |
| 29 | Fig. S59. CPL spectra of C3 PVA film collected from multiple points | S41 |

**Experimental section**

**Materials**

4-Hydroxybenzaldehyde, 1,2-dibromoethane, 1,5-dibromopentanepyrrole, 1,3-dibromopropane, poly(vinyl alcohol) (PVA), mandelic acid and potassium carbonate were purchased from TCI Chemicals. Trifluoroacetic acid (TFA), 2,3-dichloro-5,6-dicyano-1,4-benzoquinone (DDQ), potassium iodide and mercury chloride were purchased from SRL Chemicals. 2,2′-Dipicolylamine, 2-picolylamine copper chloride, cadmium chloride and zinc acetate were purchased from Sigma-Aldrich. Solvents, including methanol, DCM, chloroform, acetone, and acetonitrile, were purchased from SRL Chemicals. Anhydrous DMF, DCM is purchased from Sigma-Aldrich. All chemicals were used as received without further purifications.

**Sample Preparation for Analysis**

Freshly prepared stock solutions of samples in HPLC grade solvents with fixed concentrations were used for analysis. Samples were sonicated to ensure a maximum solubility.

**Characterization**

$^{1}H$ and $^{13}C$ NMR were measured by using a Bruker AVANCE NEO 400 MHz FT-NMR spectrometer. The $^{13}C$ NMR spectra showed low signal intensity, which can be attributed to the limited solubility of the compounds at higher concentrations in the solvent. UV–vis spectra were recorded in a quartz cuvette on an Agilent Cary-3500 UV–vis spectrophotometer. Fluorescence measurements were performed in a JASCO FP-8500 spectrofluorometer. The fluorescence lifetime was measured by using the Edinburg FLS-1000 fluorescence spectrometer using time-correlated single-photon counting (TCSPC). Absolute quantum yield measurements were performed using the integrating sphere method in the Edinburgh FLS-1000 instrument. MALDI measurement was performed using a Bruker ultraflex TOF/TOF instrument. The morphology of the nanostructures was studied using scanning electron microscope (SEM) imaging using FESEM Gemini 560, Germany, For SEM analysis, the sample solutions were coated on a silicon wafer and allowed to dry overnight.

**Preparation of PVA film**

5 wt% PVA solution was prepared by heating 200 mg of PVA in 3 mL water at 80 $^{o}C$ and was allowed to cool to room temperature. To the dissolved PVA solution, 1 mL of porphyrin-MA-Zn aggregates (93 μM) in water was added and stirred for 1 hour. The solution was then poured

onto polystyrene petri dish and allowed to dry for 3 days at room temperature to obtain self-standing polymeric film containing the nanoaggregates.

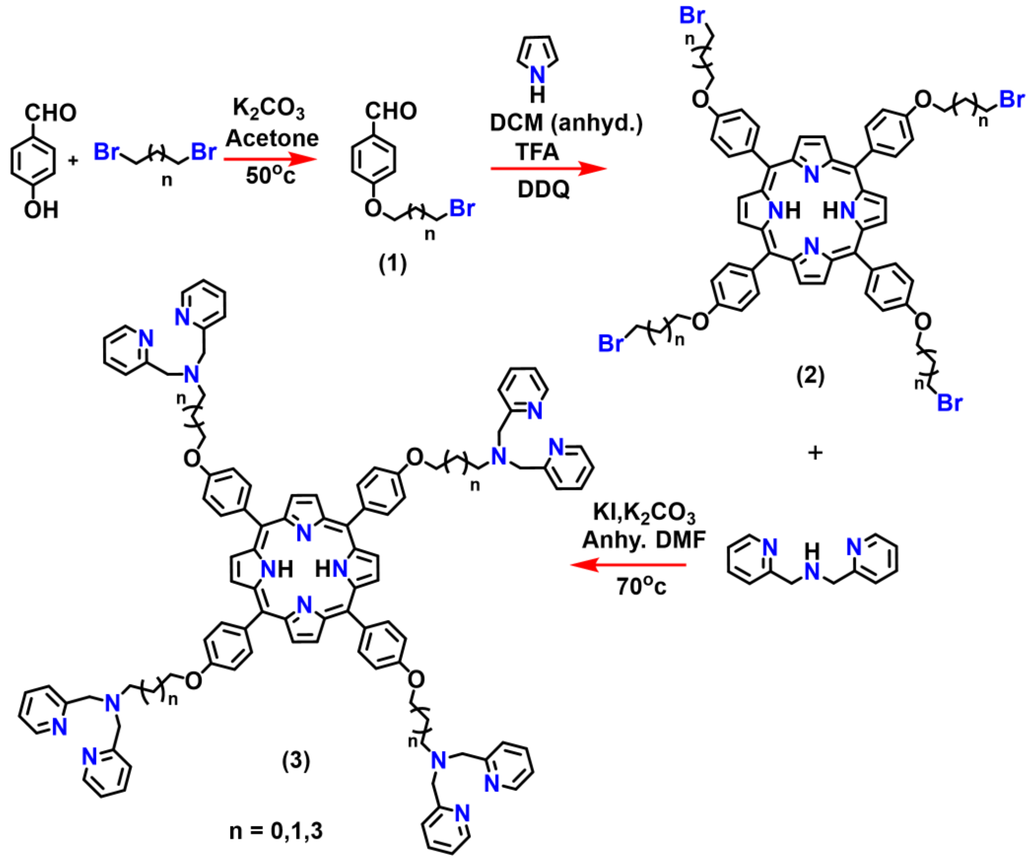


**Figure S1.** General scheme illustrating the synthesis of dipicolylamine substituted porphyrin.

i. **Synthesis of 4-(2-bromoethoxy)benzaldehyde (C2a):** A mixture of 1,2-dibromooctane (7 mL, 0.082 mol), 4-hydroxybenzaldehyde (5.0 g, 0.041 mol), and potassium carbonate (9.02 g, 0.082 mol) was added to 20 mL of DMF and heated at 75 °C with stirring overnight. Reaction progress was checked periodically by TLC. After completion, the solvent was removed under reduced pressure, and the residue was extracted with dichloromethane (DCM). The organic phase was washed with distilled water followed by brine, dried over anhydrous sodium sulfate, and filtered. Evaporation of the solvent afforded a crude product, which was purified by silica gel column chromatography (230–400 mesh) using 50% ethyl acetate in hexane. The target compound was isolated in 53% yield and its structure was verified by $^1$H NMR spectroscopy.

$^1$H NMR (400 MHz, Chloroform-*d*) δ 9.83 (s, 1H), 7.78 (d, $J$ = 8.8 Hz, 2H), 6.95 (d, $J$ = 8.8 Hz, 2H), 4.31 (t, $J$ = 6.6 Hz ,2H), 3.60 (t, $J$ = 6.6 Hz, 2H).

HRMS (ESI$^+$) m/z: calculated m/z for [$C_9H_9BrO_2$] $(M+H)^+$ - 228.9863 and $(M+H+2)^+$- 230.9843; and found 228.9854 and 230.9841.

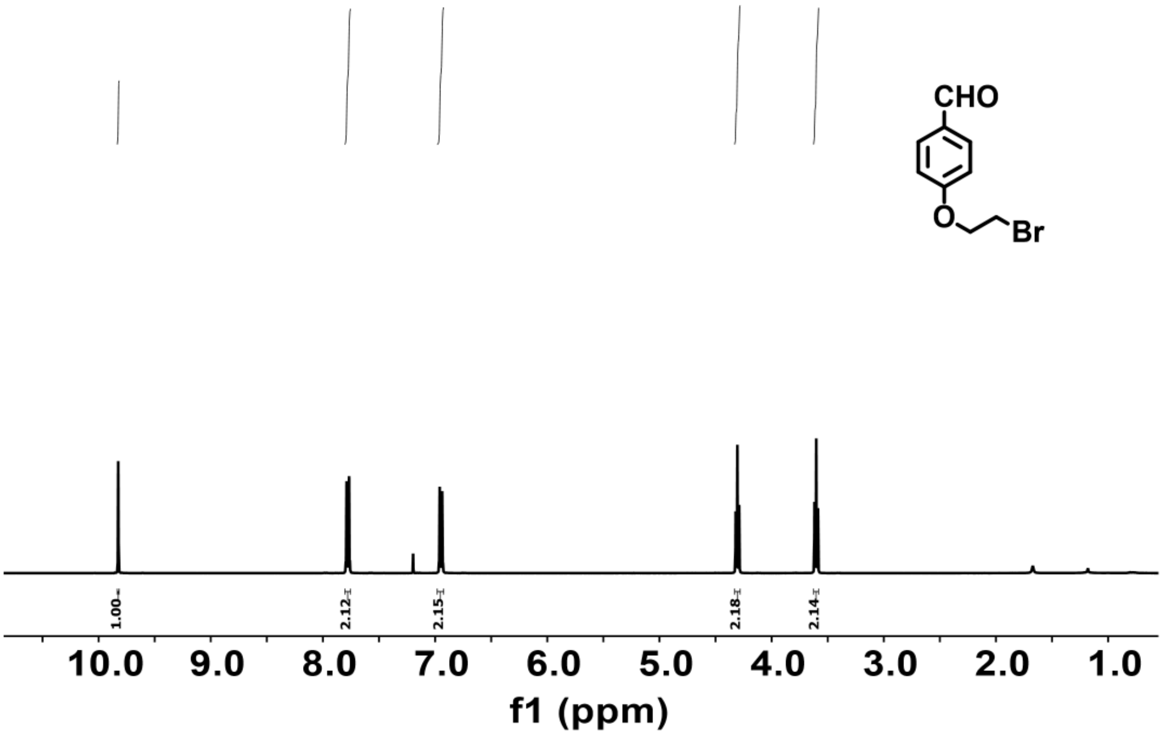


**Figure S2.** $^{1}$H NMR spectra of **C2a** in $CDCl_3$.

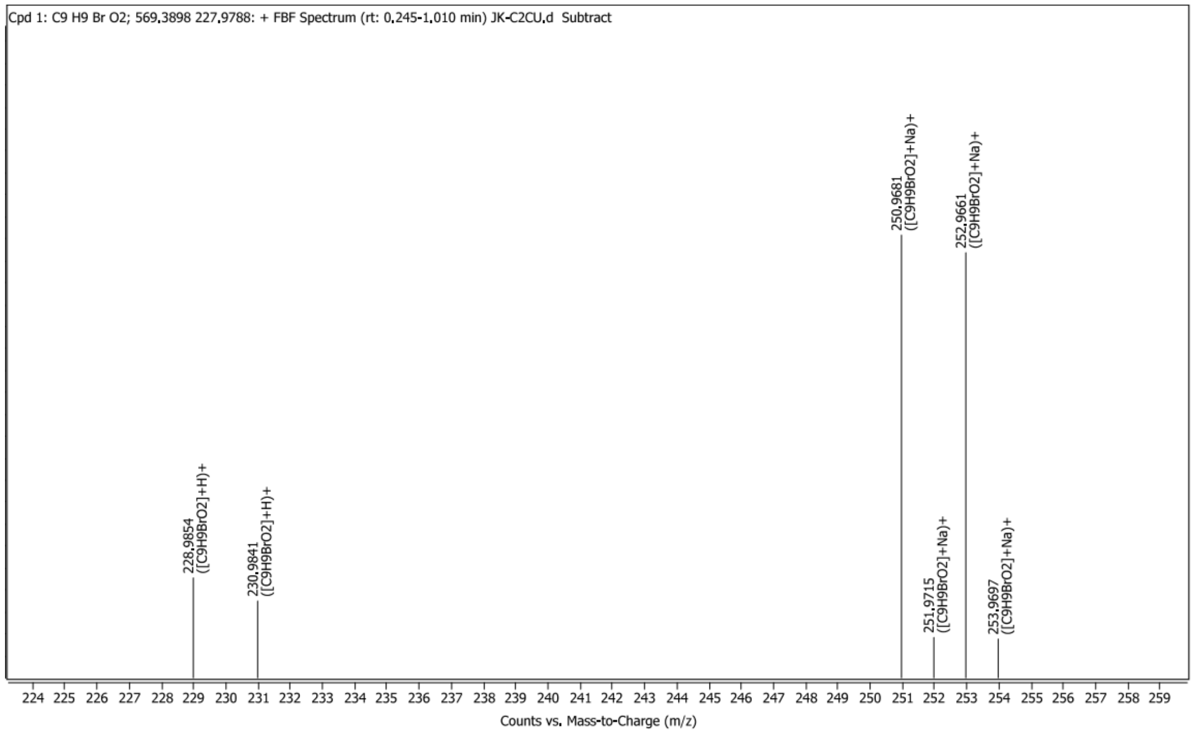


**Figure S3.** HRMS spectra of **C2a**.

ii. **Synthesis of 5,10,15,20-tetrakis(4-(2-bromoethoxy)phenyl) porphyrin (C2b)**: Under a nitrogen atmosphere, trifluoroacetic acid (TFA) (1 mL, 0.014 mol) was added to a solution of 4-(2-bromoethoxy)benzaldehyde (3.3 g, 0.014 mol) and freshly distilled pyrrole (0.969 mL, 0.014 mol) in dry DCM. The mixture was stirred at room temperature for 2 hours. DDQ (6.35 g, 0.028 mol) was then added in one portion, and the reaction was allowed to stir overnight. Reaction completion was confirmed by TLC. To neutralize excess acid, 2–3 drops of triethylamine were added to the crude mixture. After solvent removal, the residue was concentrated, and the crude solid was passed through a filtration column packed with basic alumina using a DCM/hexane solvent system to remove tarry byproducts and unreacted DDQ. The porphyrin fraction obtained after solvent evaporation was washed with methanol, affording the pure product as a bright purple solid in 13% yield. The compound was characterized by $^1$H NMR spectroscopy.

$^1$H NMR (400 MHz, Chloroform-d) δ 8.78 (s, 8H), 8.07 – 8.03 (d, $J$ = 8.7 Hz, 8H), 7.24 – 7.19 (d, $J$ = 8.7 Hz, 8H), 4.35 (t, $J$ = 4.9 Hz, 8H), 3.71 (t, $J$ = 4.9 Hz, 8H), -2.84 (s, 2H).

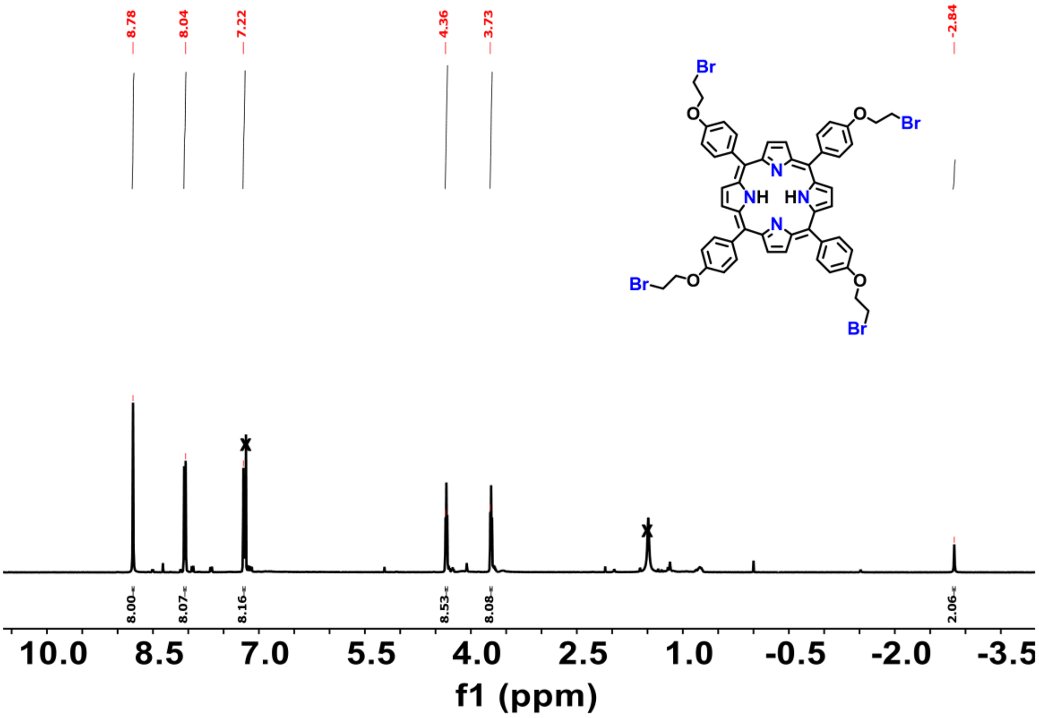


**Figure S4.** $^1$H NMR spectra of **C2b** in $CDCl_3$. Peaks marked with "x" are assigned to residual $CDCl_3$ (δ 7.26 ppm) and moisture (1.5 ppm).

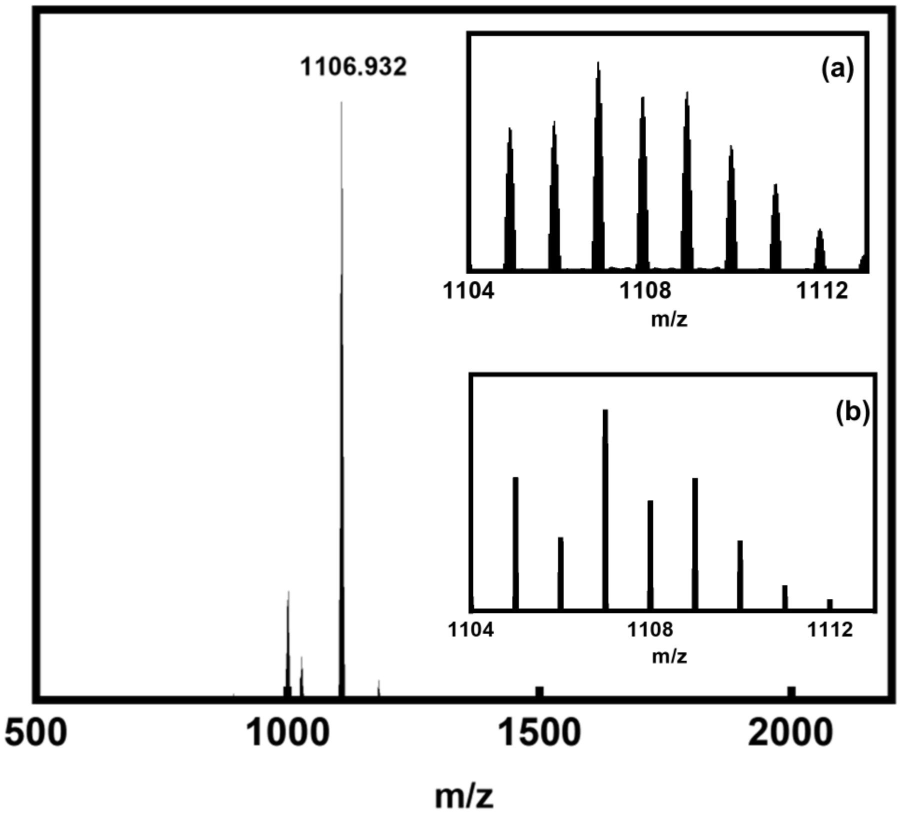


**Figure S5.** MALDI-TOF spectra of **C2b**. The inset shows an enlarged view of (a) experimental and (b) simulated mass spectrum.

iii. **Synthesis of 2,2-(Dipicolylamino)-5,10,15,20-tetrakis-(4-(2-bromoethoxy)phenyl) porphyrin (C2DP)**: A mixture of 5,10,15,20-tetrakis(4-(2-bromoethoxy)phenyl) porphyrin (100 mg, 0.09 mmol), $K_2CO_3$ (49.6 mg, 0.36 mmol) and 2,2′-dipicolylamine (0.091 mL, 0.54 mmol) was dissolved in 2 mL dry DMF. KI (7.47 mg, 0.09 mmol) dissolved in 1 mL DMF was added dropwise to the initially prepared porphyrin mixture over a period of 1 h at 75 °C. After stirring for 4 h at 75 °C, the reaction mixture was diluted with 1N HCl (15 mL) and washed twice with DCM. The aqueous layer was treated with 4N NaOH (50 mL) and extracted twice with a mixture DCM. The combined organic layers were washed with water, followed by drying over anhydrous sodium sulphate. The product was repeatedly washed with hexane for further purification to obtain the final product in 60% yield. The product was characterized using $^1$H NMR.

$^1$H NMR (400 MHz, DCM-$d_2$) δ 8.73 (s, 8H), 8.42 (d, 8H), 7.96 (d, 8H), 7.50 (s, 8H), 7.13 (d, 8H), 7.01 (d, 16H), 4.15 (m, 32H), -2.85 (s, 2H).

$^{13}C$ NMR (101 MHz, Chloroform-*d*) δ 159.16, 158.38, 157.31, 156.76, 156.52, 149.35, 136.80, 135.59, 131.93, 123.28, 123.03, 122.34, 121.63, 114.77, 112.86, 66.53, 64.34, 60.88, 53.03, 52.80, 52.46, 29.70.

HRMS (ESI$^+$) m/z: Calculated m/z for [$C_{100}H_{90}N_{16}O_4$] $(M+H)^+$ -1580.7442 and found $(M+H)^+$-1580.7374.

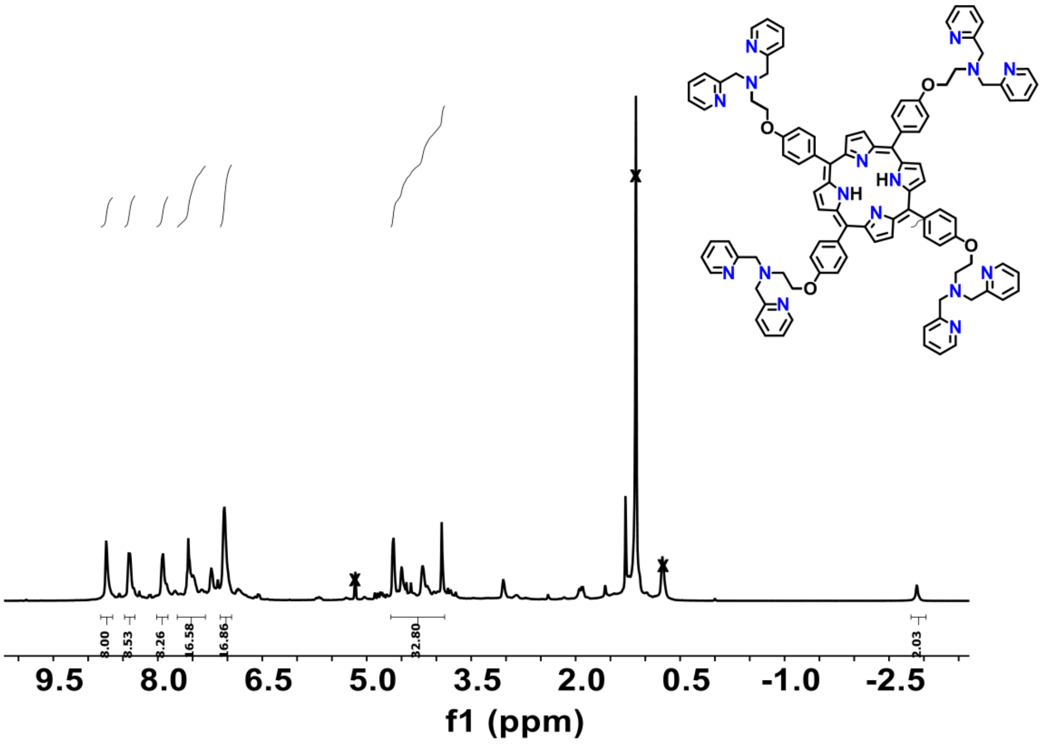


**Figure S6.** $^1H$ NMR of **C2DP** in DCM-$d_2$. Peaks marked with "x" correspond to residual DCM-$d_2$ (δ ~5.3 ppm), water in DCM-$d_2$ (δ ~1.25-1.5 ppm) and residual n-hexane (δ ~0.8 ppm).

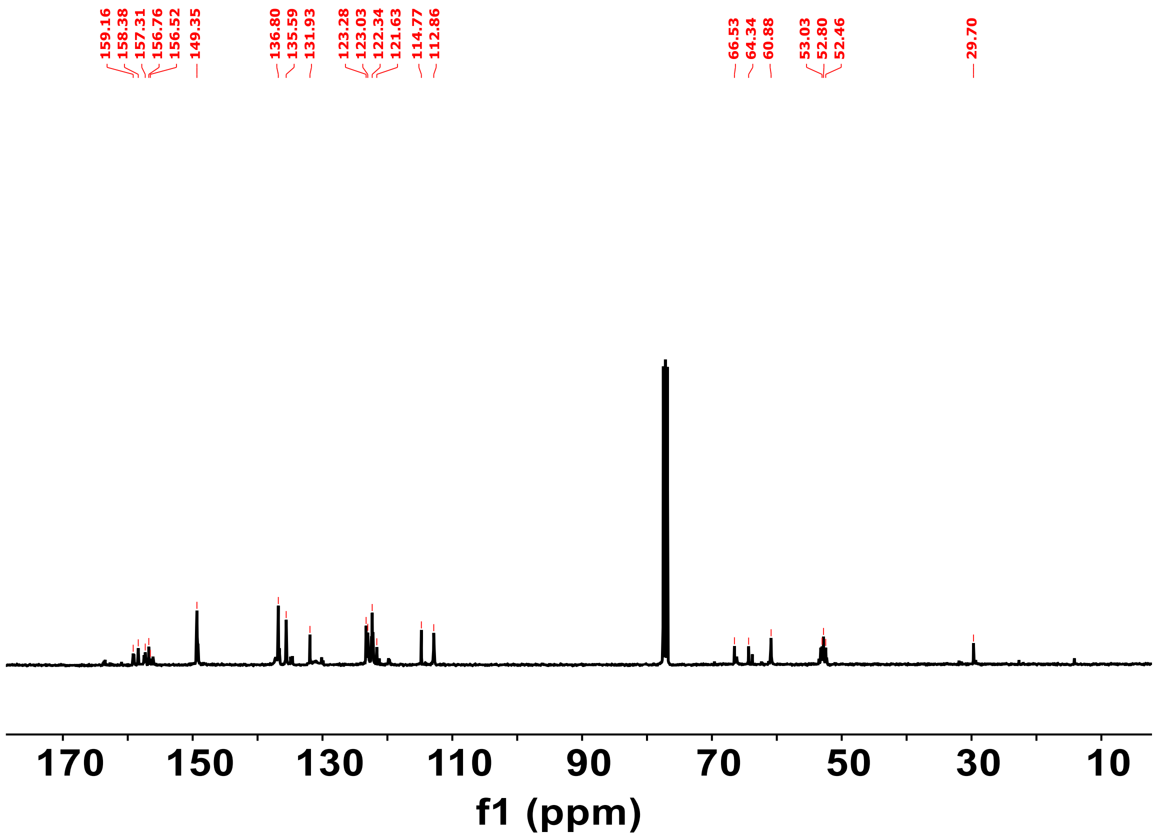


**Figure. S7.** $^{13}C$ NMR of **C2DP** in $CDCl_3$

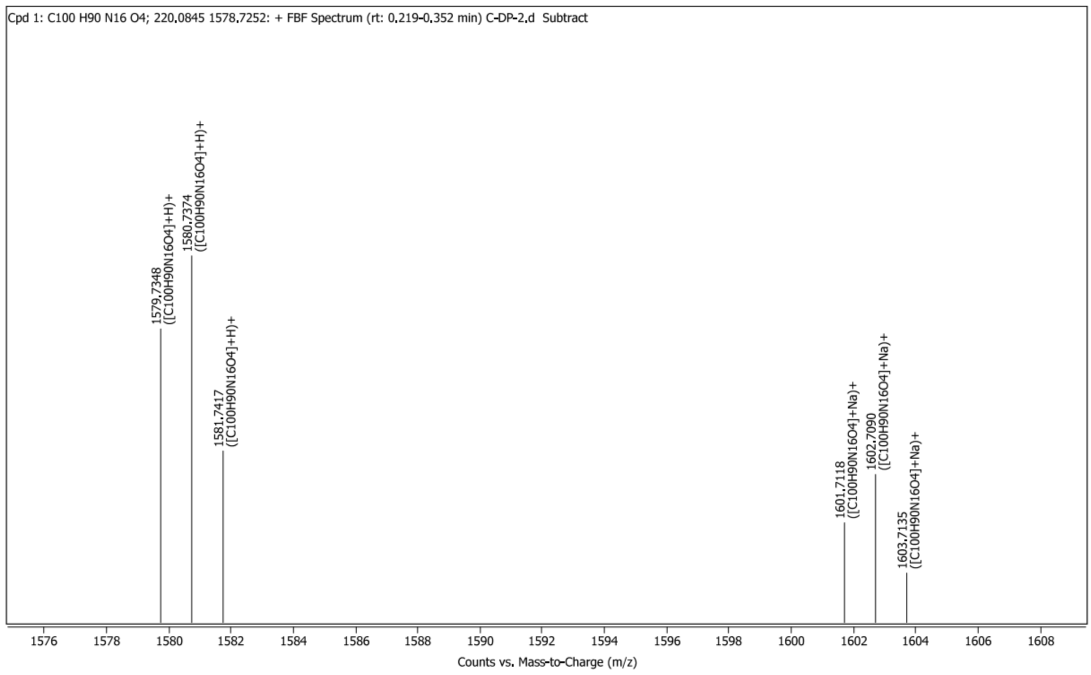


**Figure S8.** HRMS spectra of **C2DP**.

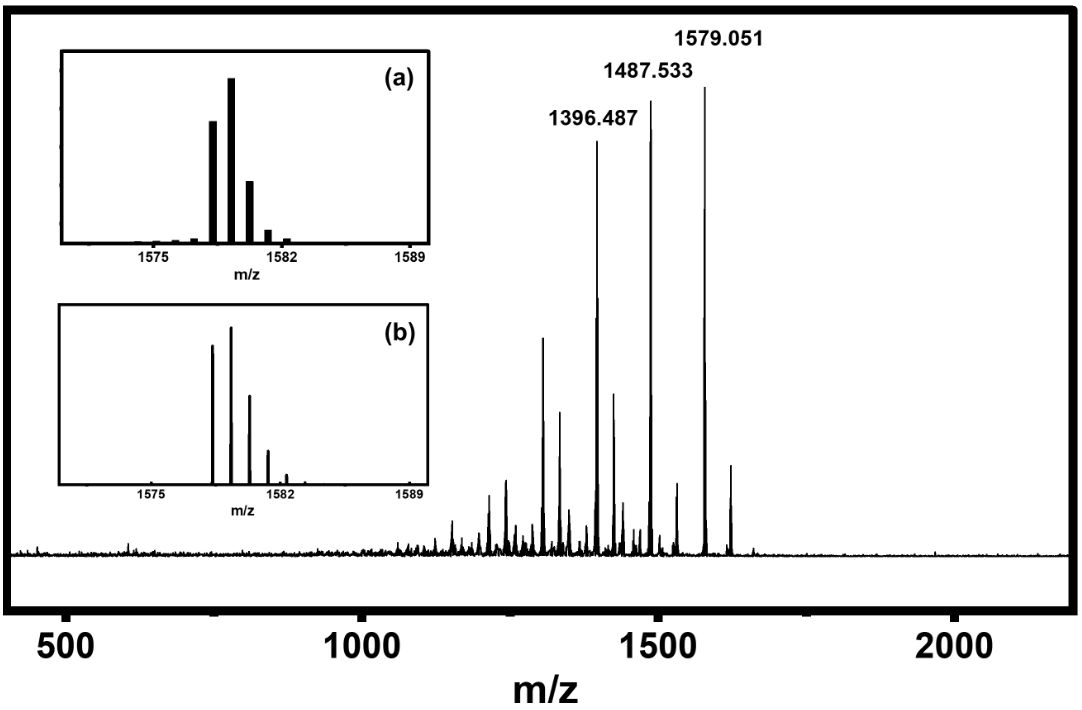


**Figure S9.** MALDI-TOF spectra of **C2DP**. The inset shows an enlarged view of (a) experimental and (b) simulated mass spectrum. Prominent fragment ions are observed at m/z 1487.533 and 1396.487, corresponding to the successive cleavage of the peripheral pyridylmethyl substituents ($\Delta m/z \approx 91$ for each fragmentation).

iv. **Synthesis of 4-(3-bromopropoxy)benzaldehyde (C3a)**: A mixture of 1,3-dibromopropane (8.07 mL, 0.08 mol), 4-hydroxybenzaldehyde (5 g, 0.04 mol), and potassium carbonate ($K_2CO_3$, 11.056g, 0.08 mol) was dissolved in 20 mL of DMF and stirred at 75 °C overnight. The progress of the reaction was monitored periodically by thin-layer chromatography (TLC). Upon completion, the solvent was removed under reduced pressure, and the resulting crude product was dissolved in dichloromethane (DCM). The solution was washed successively with brine and distilled water, and the organic layer was separated, dried over anhydrous sodium sulfate, and filtered. The solvent was removed under reduced pressure, and the crude product was purified by silica gel column chromatography (230–400 mesh) using an 40% ethyl acetate/hexane mixture as the eluent. The desired product was obtained in 51% yield and characterized by $^1H$ NMR spectroscopy.

$^1H$ NMR (400 MHz, Chloroform-*d* ) δ 9.89 (s, 1H), 7.86 – 7.82 (d, *J* = 8.8 Hz, 2H), 7.06 – 6.97 (d, *J* = 8.8 Hz, 2H), 4.20 (t, *J* = 5.8 Hz, 2H), 3.62 (t, *J* = 6.4 Hz, 2H), 2.36 (p, *J* = 6.1 Hz, 2H). HRMS (ESI+): calculated m/z for [$C_{10}H_{11}BrO_2$] $(M+Na)^+$ - 264.9839, $(M+Na+2)^+$-266.9819; found 264.9830 and 266.9809.

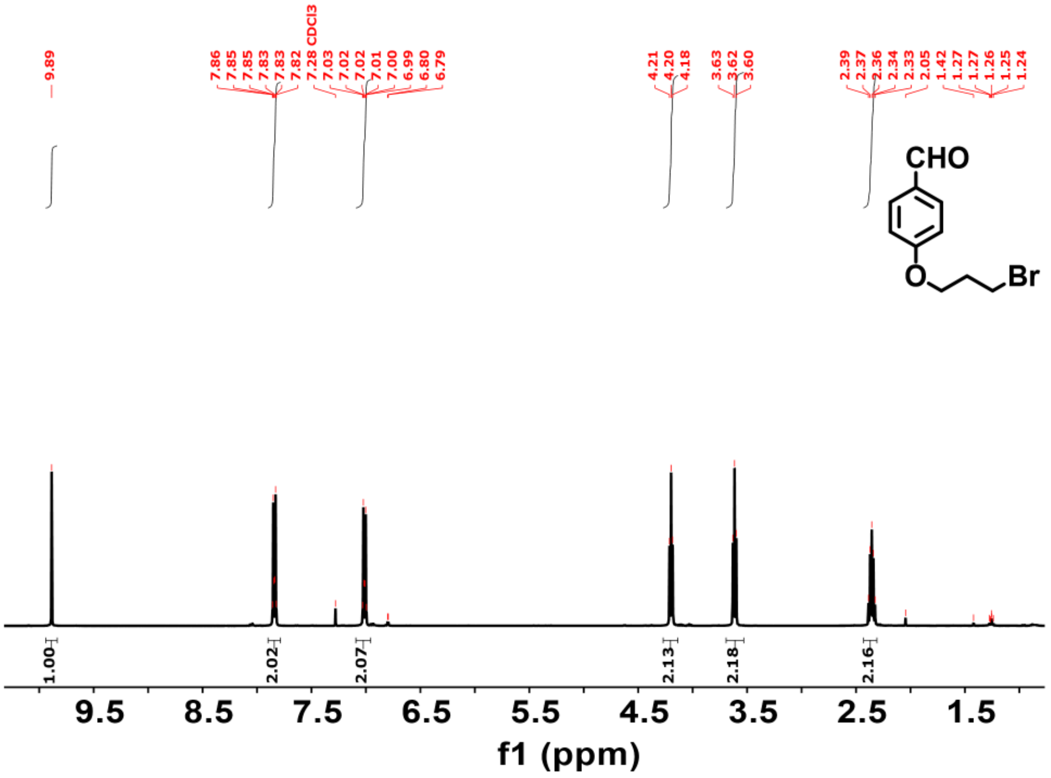


**Figure S10.** $^1H$ NMR spectra of **C3a** in $CDCl_3$.

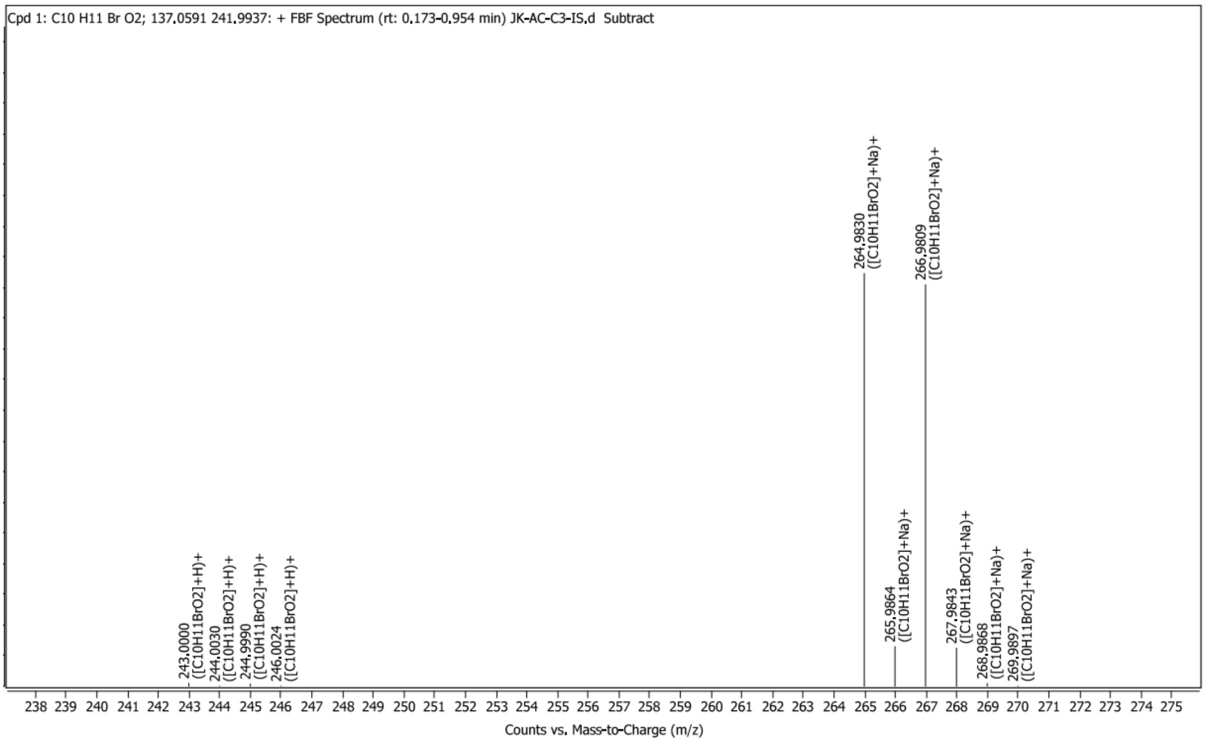


**Figure S11.** HRMS spectra of **C3a**.

v. **Synthesis of 5,10,15,20-tetrakis(4-(3-bromopropoxy)phenyl) porphyrin (C3b)**: Under a nitrogen atmosphere, trifluoroacetic acid (TFA) (0.924 mL, 0.012 mol) was added to a solution of 4-(3-bromopropoxy)benzaldehyde (3 g, 0.012 mol) and freshly distilled pyrrole (0.829 mL, 0.014 mol) in dry DCM. The mixture was stirred at room temperature for 2 hours. DDQ (5.4 g, 0.024 mol) was then added in one portion, and the reaction was allowed to stir overnight. Reaction completion was confirmed by TLC. To neutralize excess acid, 2–3 drops of triethylamine were added to the crude mixture. After solvent removal, the residue was concentrated, and the crude solid was passed through a filtration column packed with basic alumina using a DCM/hexane solvent system to remove tarry byproducts and unreacted DDQ. The porphyrin fraction obtained after solvent evaporation was washed with methanol, affording the pure product as a bright purple solid in 20% yield. The compound was characterized by $^{1}H$ NMR spectroscopy.

$^{1}H$ NMR (400 MHz, Chloroform-*d*) δ 8.90 (s, 8H), 8.16 – 8.13 (d, *J* = 8.8, 8H), 7.31 – 7.28 (d, *J* = 8.8 Hz, 8H), 4.40 (t, *J* = 5.7 Hz, 8H), 3.81 (t, *J* = 6.4 Hz, 8H), 2.57 – 2.50 (t, *J* = 6.5 Hz, 8H), -2.72 (s, 2H).

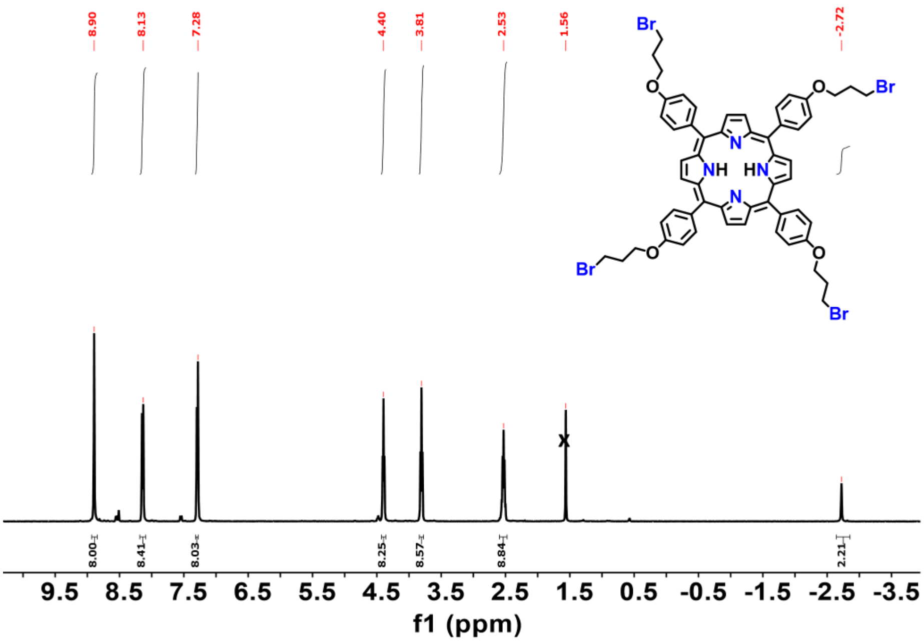


**Figure S12.** $^{1}H$ NMR spectra of **C3b** in $CDCl_3$. Peaks marked with "x" correspond to trace water in $CDCl_3$ (δ ~1.56 ppm).

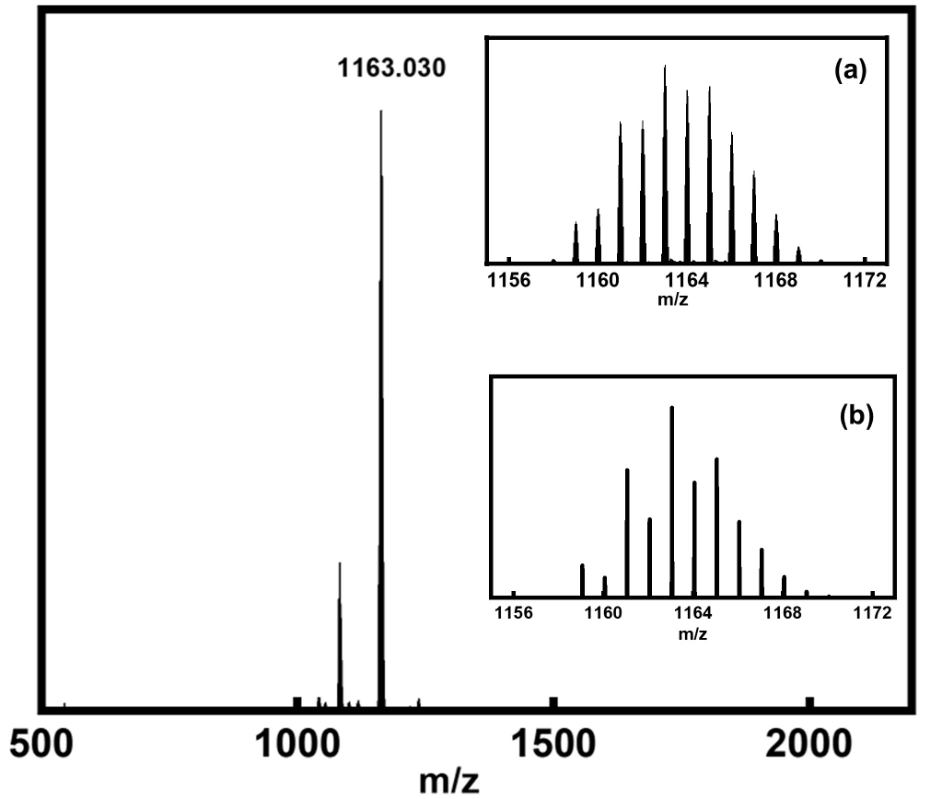


**Figure S13.** MALDI-TOF spectra of **C3b**. The inset shows an enlarged view of (a) experimental and (b) simulated mass spectrum.

vi. **Synthesis of 2,2-(Dipicolylamino)-5,10,15,20-tetrakis-(4-(3-bromopropoxy)phenyl) porphyrin (C3DP)**: A mixture of 5,10,15,20-tetrakis(4-(3-bromoethoxy)phenyl) porphyrin (100 mg, 0.086 mmol), $K_2CO_3$ (48 mg, 0.344 mmol) and 2,2′-dipicolylamine (0.092 mL, 0.516 mmol) was dissolved in 3 mL dry DMF. KI (0.014 mg, 0.086 mmol) dissolved in 2 mL DMF was added dropwise to the initially prepared porphyrin mixture over a period of 1 h at 75 °C. After stirring for 4 h at 75 °C, the reaction mixture was diluted with 1N HCl (15 mL) and washed twice with ethyl acetate. The aqueous layer was treated with 4N NaOH (50 mL) and extracted twice with DCM. The combined organic layers were washed with water, followed by drying over anhydrous sodium sulphate. The product was repeatedly washed with hexane for further purification to obtain the final product in 58% yield. The product was characterized using $^1$H NMR.

$^1$H NMR (400 MHz, DMSO-$d_6$) δ 8.86 (s, 8H), 8.53 (d, $J$ = 8.8 Hz, 8H), 8.19 – 8.01 (m, Hz, 8H), 7.78 (td, 8H), 7.62 (d, 8H), 7.32 – 7.19 (m, 16H), 4.24 (t, 8H), 3.86 (s, 16H), 2.78 (t, $J$ = 6.7 Hz, 8H), 2.16 – 2.03 (m, 8H), -2.87 (s, 2H).

$^{13}C$ NMR (101 MHz, Chloroform-*d*) δ 158.11, 157.61, 156.37, 155.90, 148.22, 135.71, 134.50, 133.37, 122.14, 121.82, 121.23, 119.89, 119.79, 118.79, 111.68, 64.99, 61.8, 59.33, 52.54, 50.17, 28.64, 21.64, 13.10.

HRMS ESI (+): m/z calculated for [$C_{104}H_{98}N_{16}O_4$] $[M+H]^+$: 1636.8068; found $(M+H)^+$ - 1636.8025.

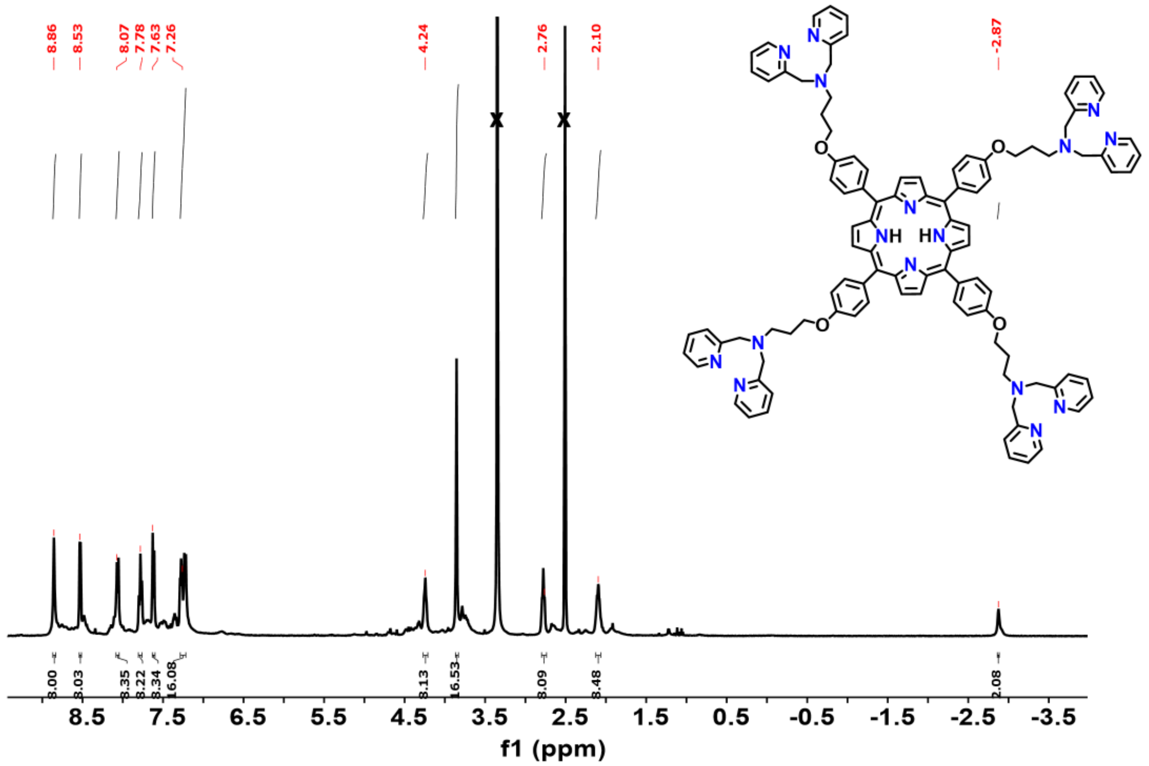


**Figure S14.** $^1H$ NMR spectra of **C3DP** in DMSO-d6. Peaks marked with "x" correspond to residual DMSO-d (δ 2.50 ppm), trace water in DMSO-$d_6$ (δ ~3.33 ppm).

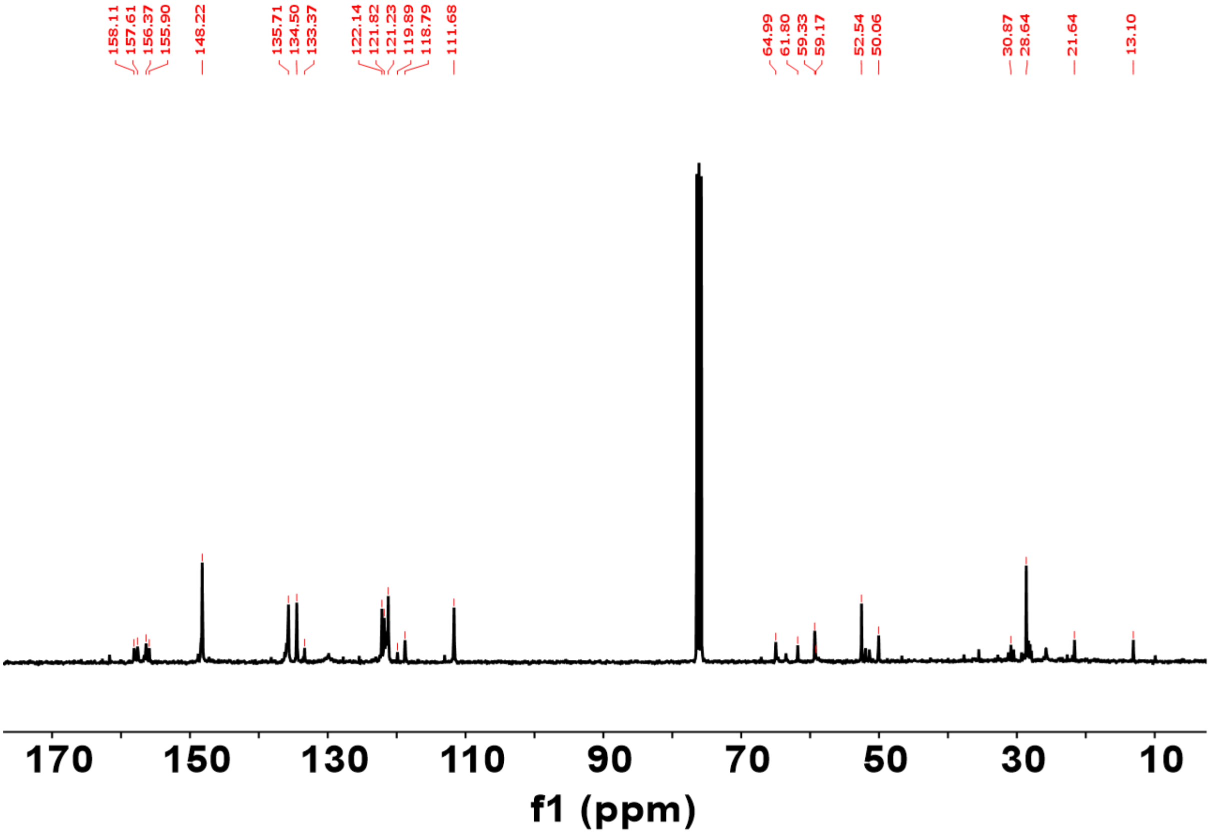


**Figure S15.** $^{13}$C NMR spectra of **C3DP** in $CDCl_3$.

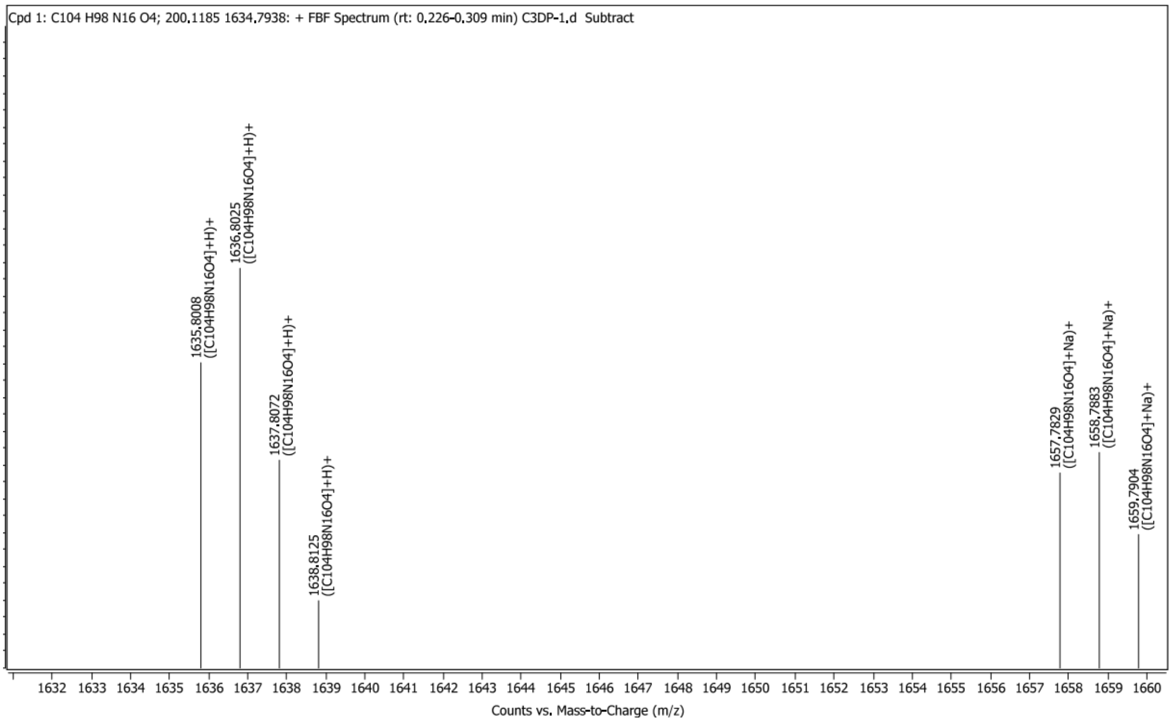


**Figure S16.** HRMS spectra of **C3DP**.

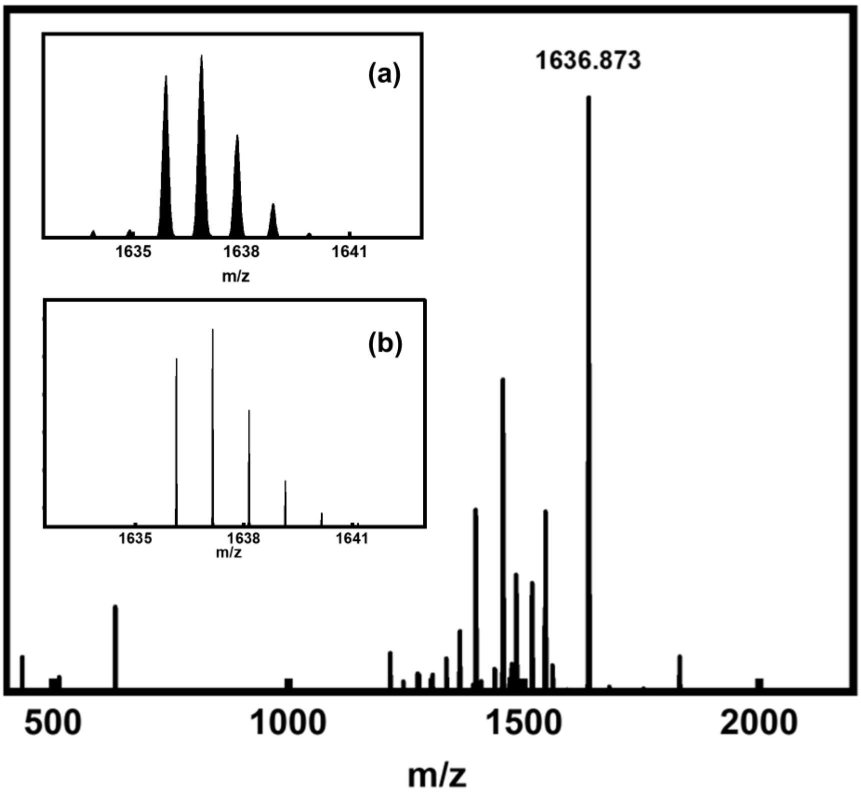


**Figure S17.** MALDI-TOF spectra of **C3DP**. The inset shows an enlarged view of (a) experimental and (b) simulated mass spectrum.

vii. **Synthesis of 2-(Picolylamino)-5,10,15,20-tetrakis-(4-(3-bromopropoxy)phenyl) porphyrin (C3P)**: A mixture of 5,10,15,20-tetrakis(4-(3-bromoethoxy)phenyl) porphyrin (100 mg, 0.086 mmol), $K_2CO_3$ (48 mg, 0.344 mmol) and 2-Picolylamine (0.052 mL, 0.516 mmol) was dissolved in 3 mL dry DMF. KI (0.014 mg, 0.086 mmol) dissolved in 2 mL DMF was added dropwise to the initially prepared porphyrin mixture over a period of 1 h at 75 °C. After stirring for 4 h at 75 °C, the reaction mixture was diluted with 1N HCl (15 mL) and washed twice with DCM. The aqueous layer was treated with 4N NaOH (50 mL) and extracted twice with DCM. The combined organic layers were washed with water, followed by drying over anhydrous sodium sulphate. The product was repeatedly washed with hexane for further purification to obtain the final product in 55% yield. The product was characterized using 1H NMR.

$^1$H NMR (400 MHz, Chloroform-*d*) δ 8.79 (s, 8H), 8.51 (d, *J* = 8.7 Hz, 8H), 8.02 (d, *J* = 8.7 Hz, 8H), 7.62 (d, *J* = 7.8 Hz, 8H), 7.10 (m, 8H), 4.72 (t, 8H), 4.45 (m, 16H), 4.21 (m, 8H), -2.78 (s, 2H).

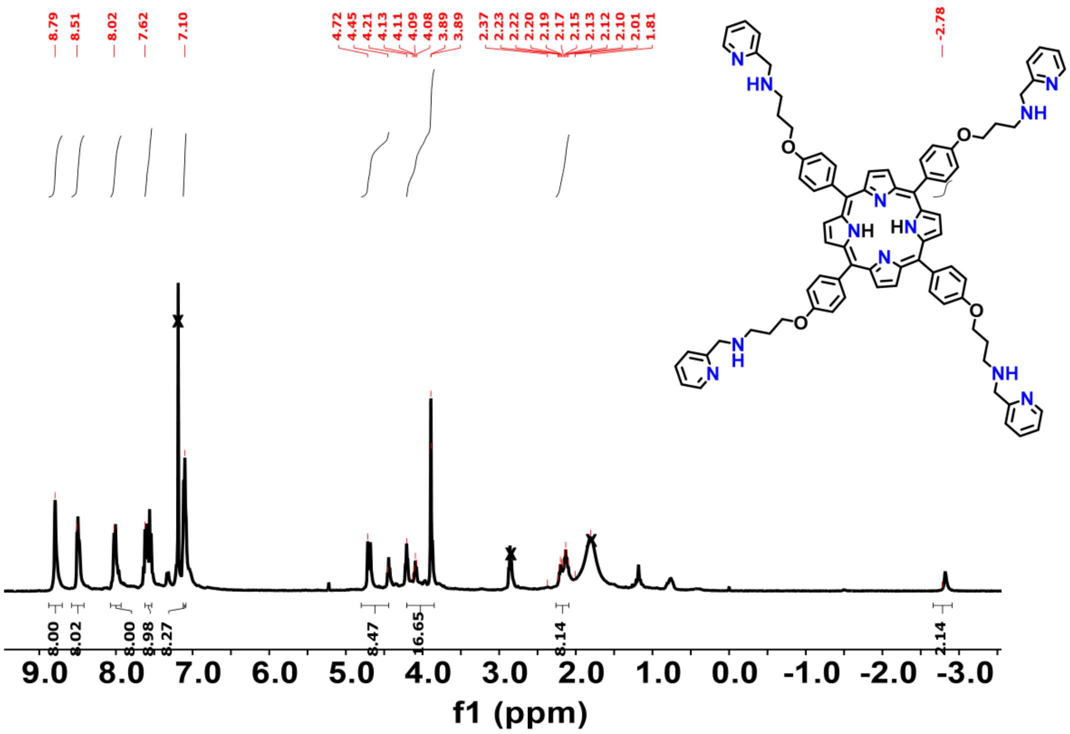


**Figure S18.** $^{1}$H NMR spectra of **C3P** in $CDCl_3$. Peaks marked with "x" are assigned to residual $CDCl_3$ (δ 7.26 ppm), trace water in $CDCl_3$ (δ ~1.56 ppm), and trace DMF (δ ~2.75–2.95 ppm).

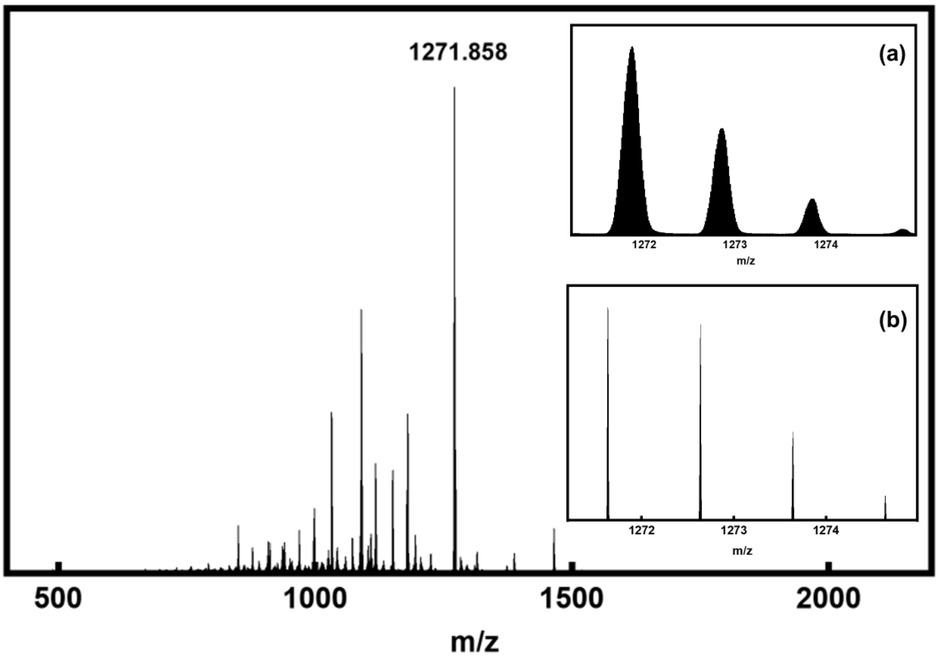


**Figure S19.** (a) Experimental and (b) simulated spectra of MALDI-TOF spectra of **C3P**. The inset shows an enlarged view of the mass spectrum.

viii. **Synthesis of 4-(5-bromopentoxy)benzaldehyde (C5a)**: A mixture of 1,5-dibromopentane (11 mL, 0.08 mol), 4-hydroxybenzaldehyde (5 g, 0.04 mol), and potassium carbonate ($K_2CO_3$, 11.05 g, 0.08 mol) was dissolved in 20 mL of DMF and stirred at 75 °C overnight. The progress of the reaction was monitored periodically by thin-layer chromatography (TLC). Upon completion, the solvent was removed under reduced pressure, and the resulting crude product was dissolved in dichloromethane (DCM). The solution was washed successively with brine and distilled water, and the organic layer was separated, dried over anhydrous sodium sulfate, and filtered. The solvent was removed under reduced pressure, and the crude product was purified by silica gel column chromatography (230–400 mesh) using an 20% ethyl acetate/hexane mixture as the eluent. The desired product was obtained in 55% yield and characterized by $^1$H NMR spectroscopy.

$^1$H NMR (400 MHz, Chloroform-*d*) δ 9.84 (s, 1H), 7.88 – 7.72 (d, *J* = 8.7 Hz, 2H), 6.96 (d, *J* = 8.5 Hz, 2H), 4.02 (t, *J* = 6.3 Hz, 2H), 3.42 (t, *J* = 6.7 Hz, 2H), 1.86 (m, 4H), 1.66 – 1.56 (m, 2H).

HRMS (ESI+): calculated m/z for [$C_{12}H_{15}BrO_2$] $(M+H)^+$ - 271.0333 and $(M+H+2)^+$-273.0313; found 271.0316 and 273.0302.

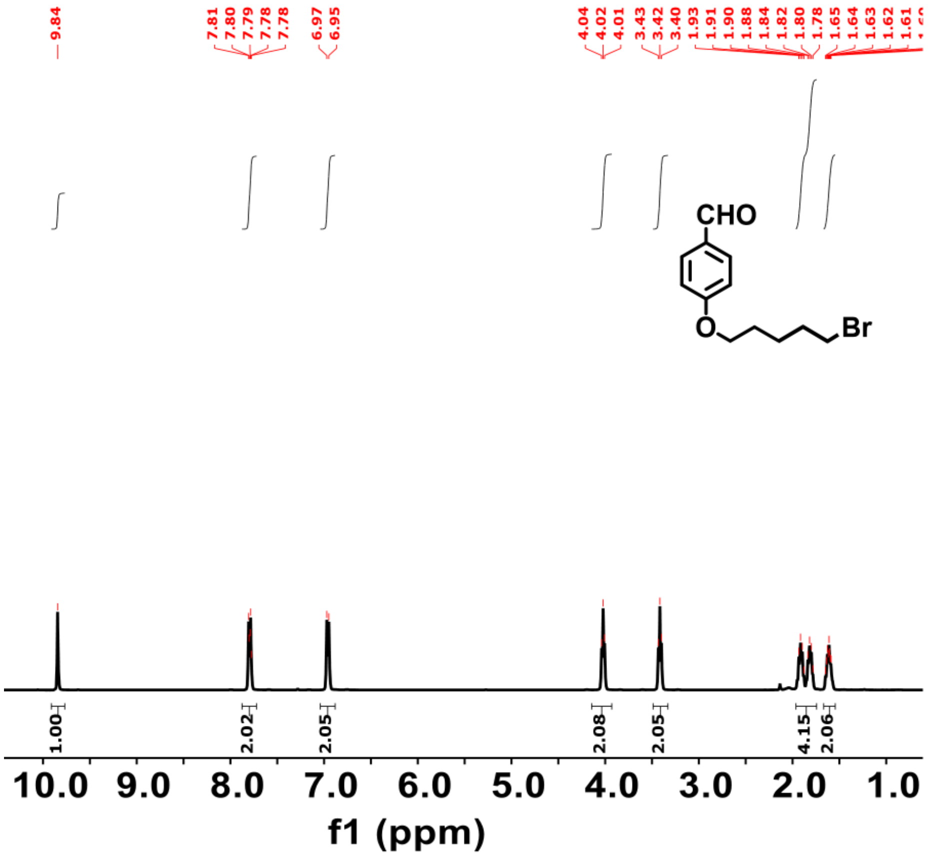


**Figure S20.** $^{1}$H NMR spectra of **C5a** in $CDCl_3$.

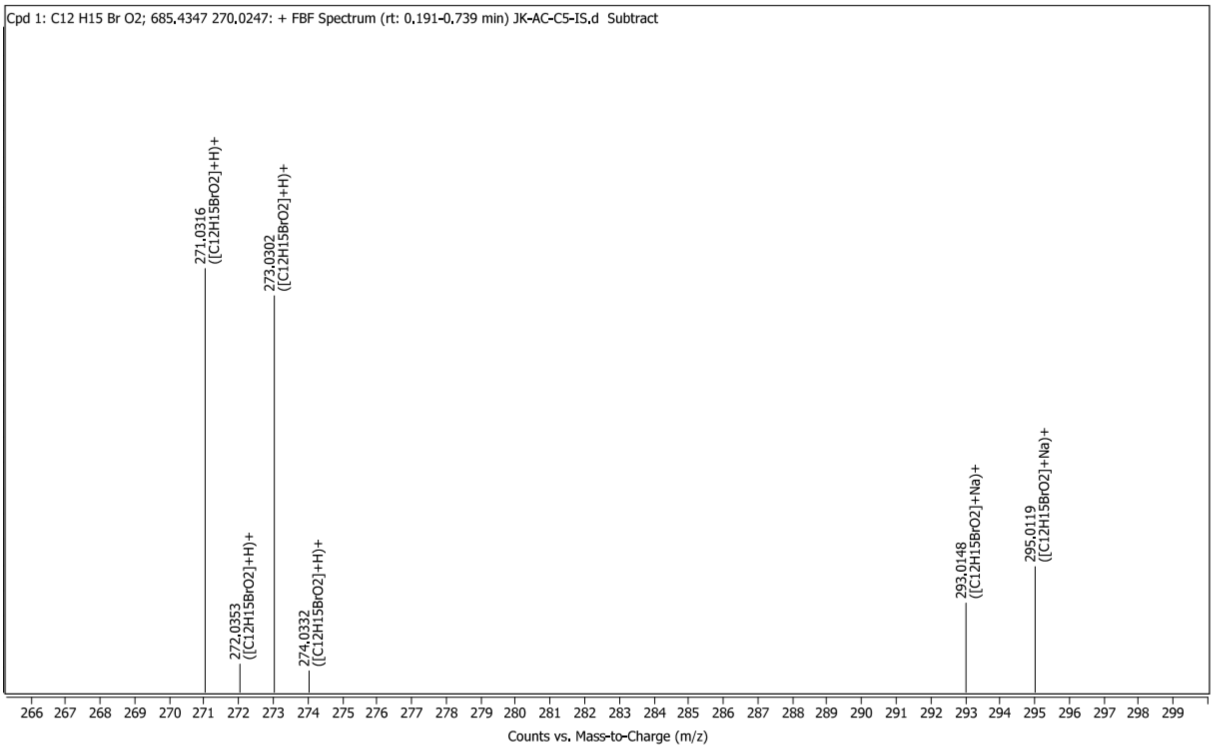


**Figure S21.** HRMS spectra of **C5a**.

ix. **Synthesis of 5,10,15,20-tetrakis(4-(5-bromopentoxy)phenyl) porphyrin (C5b)**: Under a nitrogen atmosphere, trifluoroacetic acid (TFA) (0.847 mL, 0.011 mol) was added to a solution of 4-(5-bromopentoxy)benzaldehyde (3 g, 0.011 mol) and freshly distilled pyrrole (0.737 mL, 0.011 mol) in dry DCM. The mixture was stirred at room temperature for 2 hours. DDQ (5 g, 0.022 mol) was then added in one portion, and the reaction was allowed to stir overnight. Reaction completion was confirmed by TLC. To neutralize excess acid, 2–3 drops of triethylamine were added to the crude mixture. After solvent removal, the residue was concentrated, and the crude solid was passed through a filtration column packed with basic alumina using a DCM/hexane solvent system to remove tarry byproducts and unreacted DDQ. The porphyrin fraction obtained after solvent evaporation was washed with methanol, affording the pure product as a bright purple solid in 15% yield. The compound was characterized by $^{1}$H NMR spectroscopy.

$^{1}$H NMR (400 MHz, Chloroform-*d*) δ 8.79 (s, 8H), 8.04 (d, $J$ = 8.5 Hz, 8H), 7.20 (d, $J$ = 8.5 Hz, 8H), 4.20 (t, $J$ = 6.3 Hz, 8H), 3.47 (t, $J$ = 6.8 Hz, 8H), 2.03 – 1.92 (m, 16H), 1.75 (d, $J$=6.5 Hz, 8H), -2.82 (s, 2H).

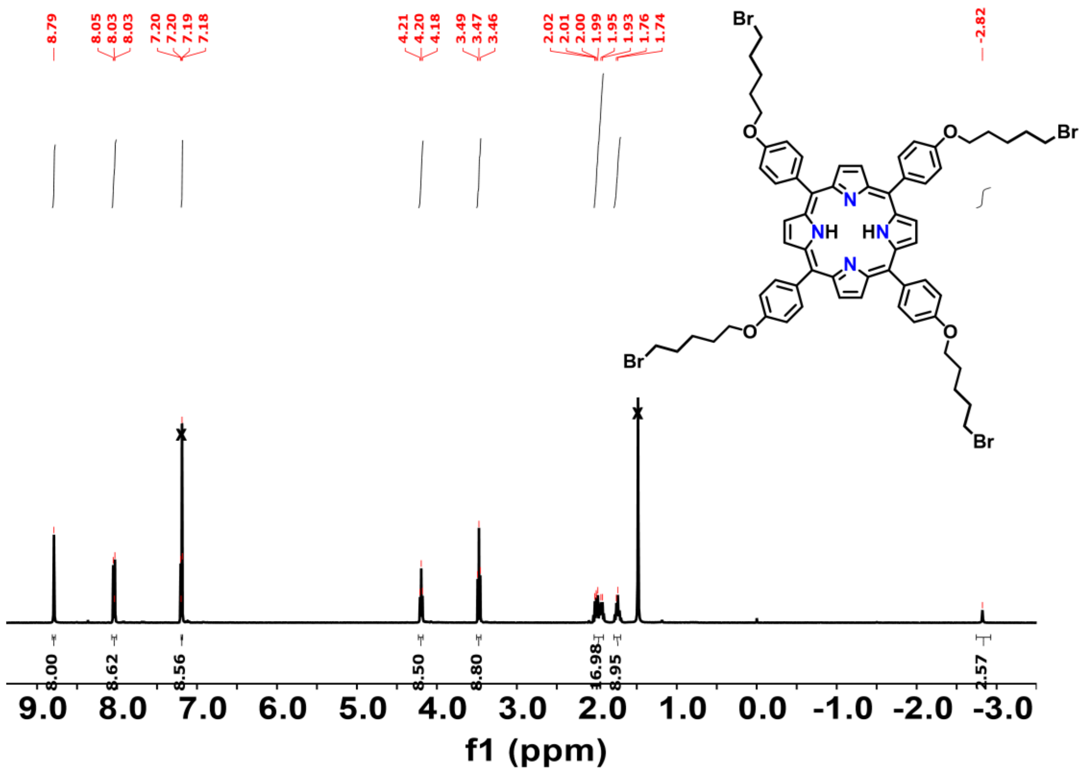


**Figure S22.** $^{1}$H NMR spectra of **C5b** in $CDCl_3$. Peaks marked with "x" are assigned to residual $CDCl_3$ (δ 7.26 ppm) and trace water in $CDCl_3$ (δ ~1.56 ppm)

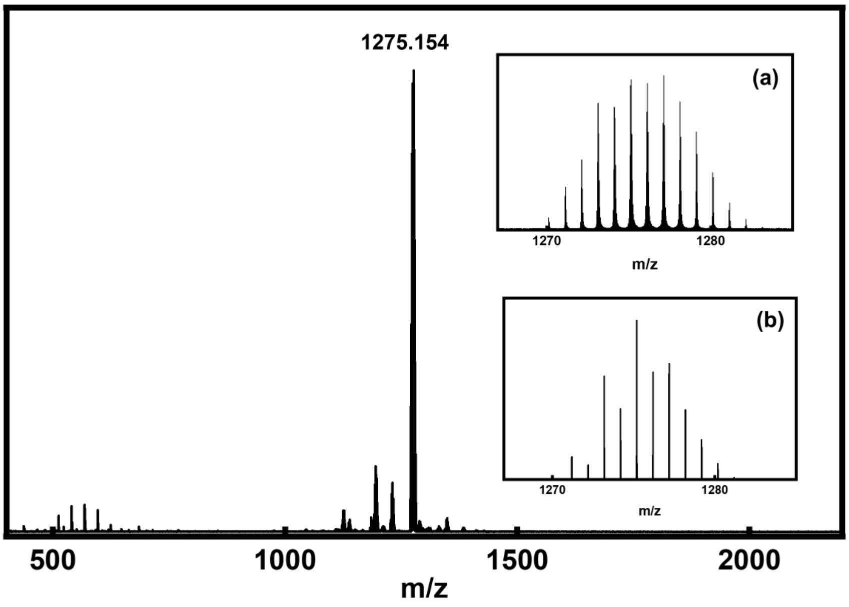


**Figure S23.** MALDI-TOF spectra of **C5b**. The inset shows an enlarged view of (a) experimental and (b) simulated mass spectrum.

x. **Synthesis of 2,2'-(Dipicolylamino)-5,10,15,20-tetrakis-(4-(5-bromopentoxy)phenyl) porphyrin (C5DP)**: A mixture of 5,10,15,20-tetrakis(4-(5-bromopentoxy)phenyl) porphyrin (100 mg, 0.00693 mmol), $K_2CO_3$ (48.4mg, 0.00035 mol) and 2,2′-dipicolylamine (0.074 mL, 0.04158 mmol) was dissolved in 3 mL dry DMF. KI (58.1 mg, 0.00035 mmol) dissolved in 2 mL DMF was added dropwise to the initially prepared porphyrin mixture over a period of 1 h at 75 °C. After stirring for 4 h at 75 °C, the reaction mixture was diluted with 1N HCl (15 mL) and washed twice with DCM. The aqueous layer was treated with 4N NaOH (50 mL) and extracted twice with DCM. The combined organic layers were washed with water, followed by drying over anhydrous sodium sulphate. The product was repeatedly washed with hexane for further purification to obtain the final product in 58% yield. The product was characterized using $^1$H NMR.

$^1$H NMR (400 MHz, DMSO-$d_6$) δ 8.86 (s, 8H), 8.55 (d, $J$ = 8.1 Hz, 8H), 8.10 (d, $J$ = 8.1 Hz, 8H), 7.90 – 7.40 (m, 16H), 7.25 (m, 8H), 7.11 (m, 8H), 4.74 (d, 16H), 4.22 (ddt, 8H), 3.46 (s, 16H), 1.80 – 1.72 (m, 16H), -2.74 (s, 2H).

$^{13}$C NMR (101 MHz, Chloroform-*d*) δ 165.87, 161.65, 160.2, 157.81, 156.04, 148.31, 147.31, 136.27, 136.03, 135.69, 134.56, 125.47, 124.11, 121.33, 111.69, 66.91, 64.82, 52.58, 35.53, 30.87, 30.45, 28.64, 28.31, 21.64, 13.10.

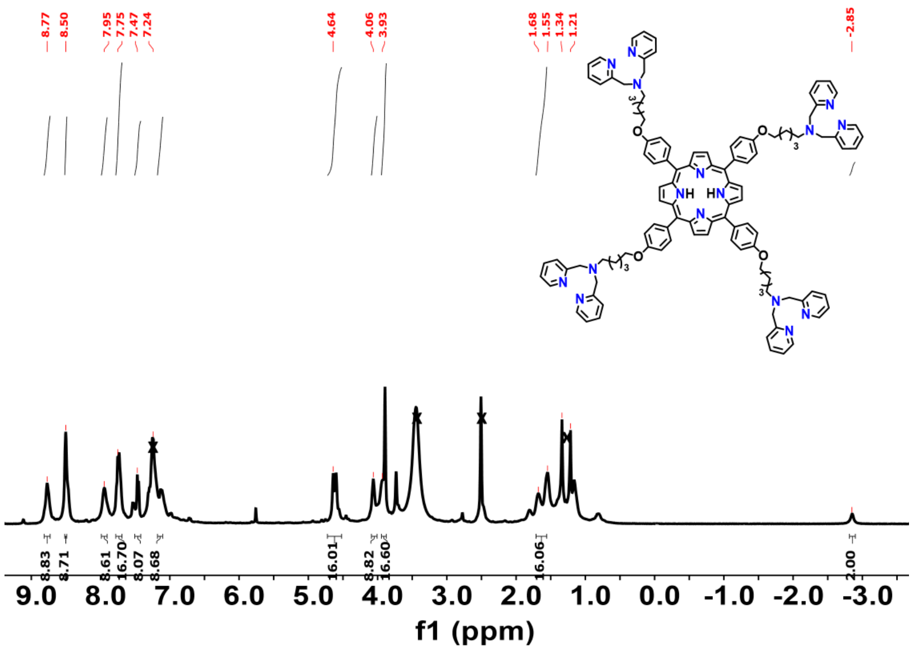


**Figure S24.** $^{1}H$ NMR spectra of **C5DP** in DMSO-d₆. Peaks marked with "x" correspond to residual DMSO-d (δ~2.50 ppm), trace water in DMSO-d₆ (δ ~3.33 ppm), trace DMF (δ ~7.5ppm) and trace *n*-pentane (δ ~1.27 ppm).

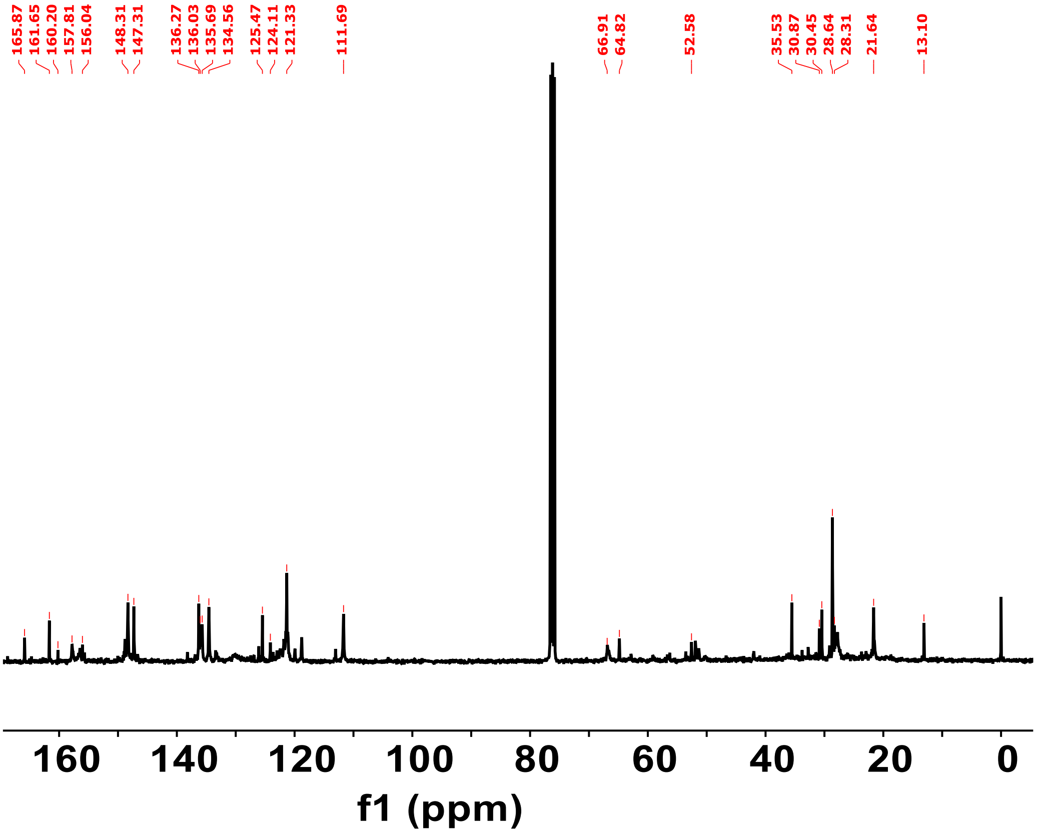


**Figure S25.** $^{13}C$ NMR spectra of **C5DP** in $CDCl_3$.

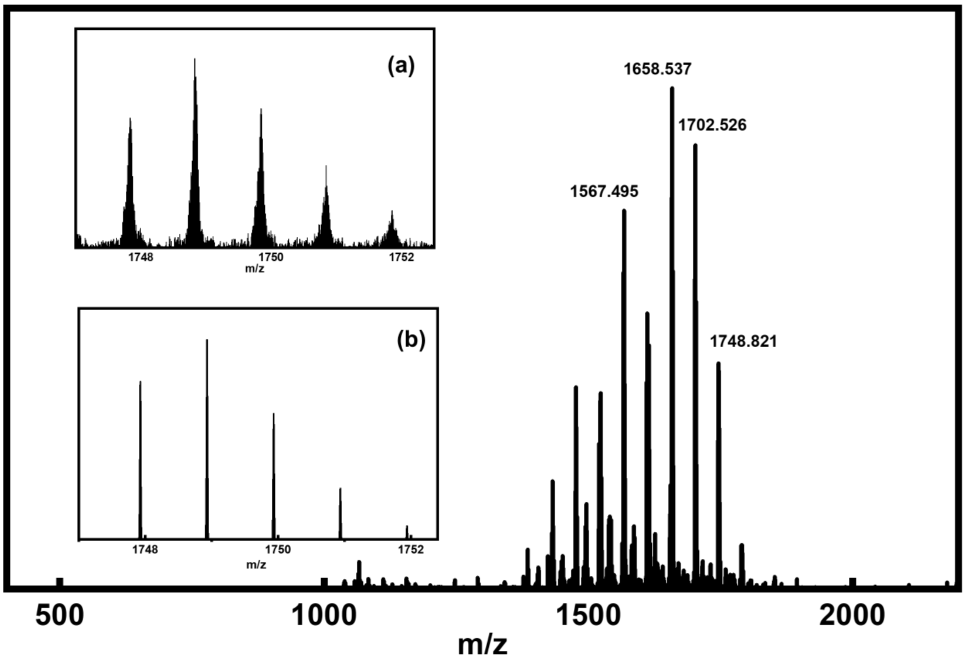


**Figure S26.** (a) Experimental and (b) simulated MALDI-TOF spectra of **C5DP**. The inset shows an enlarged view of the mass spectrum. Prominent fragment ions are observed at *m/z* 1658.537 and 1567.495, corresponding to the successive loss of one and two peripheral pyridylmethyl substituents ($\Delta m/z \approx 91$ for each fragmentation).

xi. **Synthesis of 4-(8-bromoctoxy)benzaldehyde (C8a)**: A mixture of 1,8-dibromooctane (12 mL, 0.0654 mol), 4-hydroxybenzaldehyde (4 g, 0.0327 mol), and $K_2CO_3$ (9.02 g, 0.0654 mol) was dissolved in 20 mL of acetone and stirred overnight at 50 °C. The progress of the reaction was monitored by TLC. Once the reaction was complete, the solvent was removed under reduced pressure. The crude product was dissolved in DCM and washed several times with brine and distilled water. The organic layer was then dried over anhydrous sodium sulfate and filtered. After solvent removal, the residue was purified by silica gel column chromatography (230–400 mesh) using 8% ethyl acetate/hexane as the eluent, yielding the product in 53% yield. The purified compound was characterized by 1H NMR spectroscopy.

$^1$H NMR (400 MHz, Chloroform-*d*) δ 9.74 (s, 1H), 7.69 (d, *J* = 8.3 Hz, 2H), 6.86 (d, *J* = 8.4 Hz, 2H), 3.89 (t, *J* = 6.5 Hz, 2H), 3.27 (t, 2H), 1.70 (m, 4H), 1.38 – 1.18 (m, 8H).

HRMS (ESI+): m/z Calculated for [$C_{15}H_{21}BrO_2$] $(M+Na)^+$-335.0616 and $(M+2+Na)^+$-337.0596 and found 335.0610 and 337.0591.

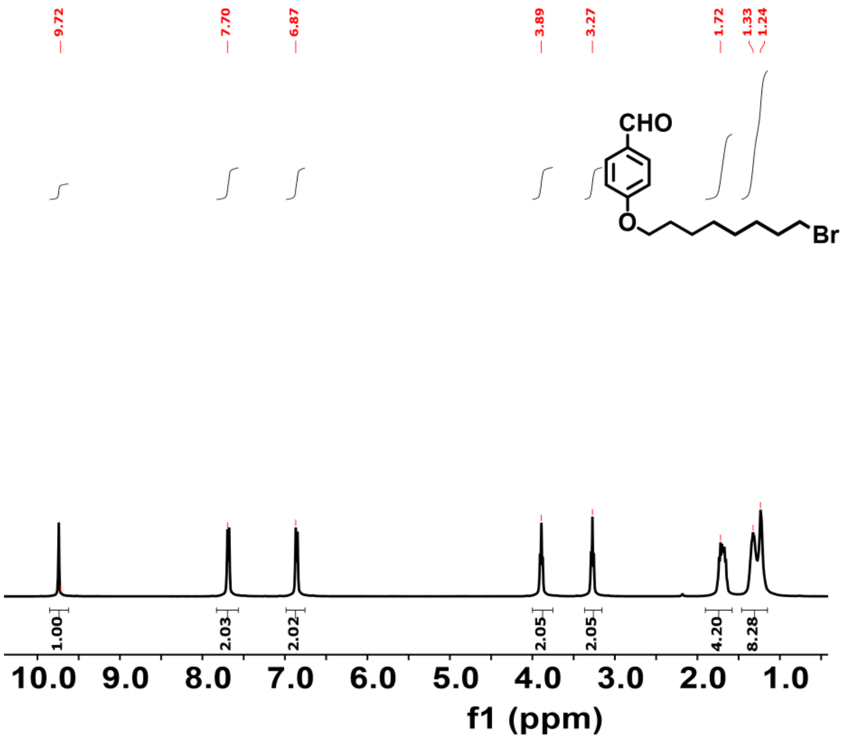


**Figure S27.** $^{1}$H NMR spectra of **C8a** in $CDCl_3$.

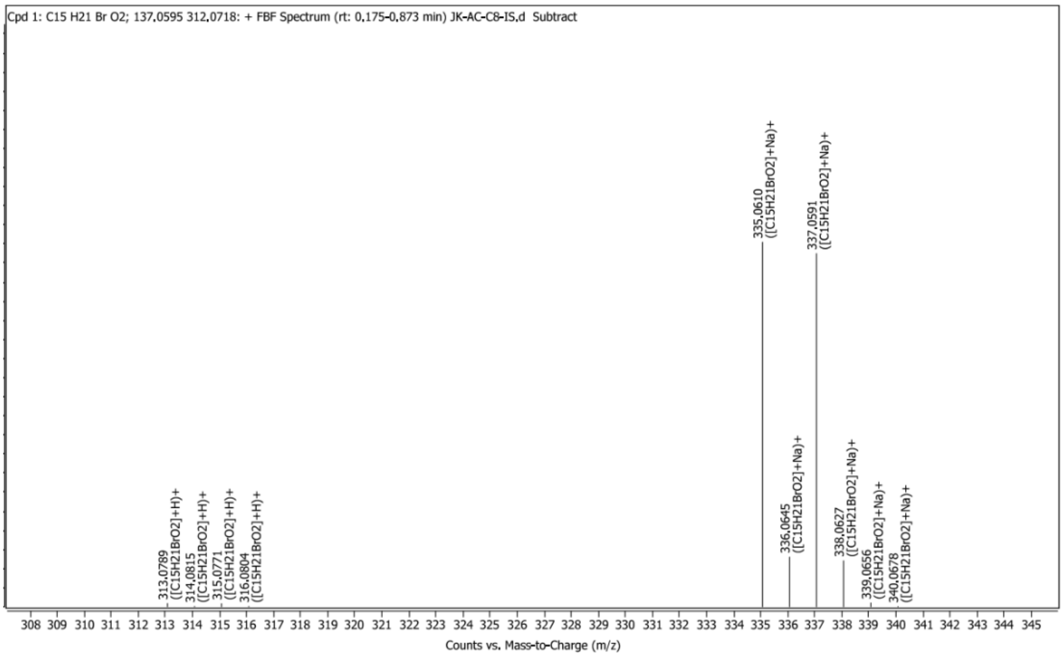


**Figure S28.** HRMS spectra of **C8a**.

xii. **Synthesis of 5,10,15,20-tetrakis(4-(8-bromoctoxy)phenyl) porphyrin (C8b)**: Under a nitrogen atmosphere, trifluoroacetic acid (TFA) (0.735 mL, 0.00961 mol) was added to a solution of 4-(8-bromooctoxy)benzaldehyde (3 g, 0.00961 mol) and freshly distilled pyrrole (0.66 mL, 0.00961 mol) in dry DCM. The mixture was stirred at room temperature for 2 h. DDQ (3.385 g, 0.0144 mol) was then added in one portion, and the reaction was allowed to proceed overnight. Reaction completion was confirmed by TLC. To quench the excess acid, 2–3 drops of triethylamine were introduced. The solvent was removed under reduced pressure, and the resulting crude solid was subjected to filtration through a basic alumina column using a DCM/hexane solvent system to eliminate tar-like impurities and excess DDQ. The porphyrin fraction obtained after solvent removal was washed with methanol, yielding the pure product as a bright purple solid in 13% yield. The compound was characterized by $^{1}H$ NMR spectroscopy.

$^{1}H$ NMR (400 MHz, Chloroform-*d*) δ 8.86 (s, 8H), 8.09 (d, $J$ = 8.1 Hz, 8H), 7.24 – 7.21 (m, 8H), 4.17 (dd, 8H), 3.44 (t, $J$ = 6.8 Hz, 8H), 1.92 (dt, 16H), 1.59 (t, $J$ = 7.7 Hz, 32H), -2.72 (s, 2H).

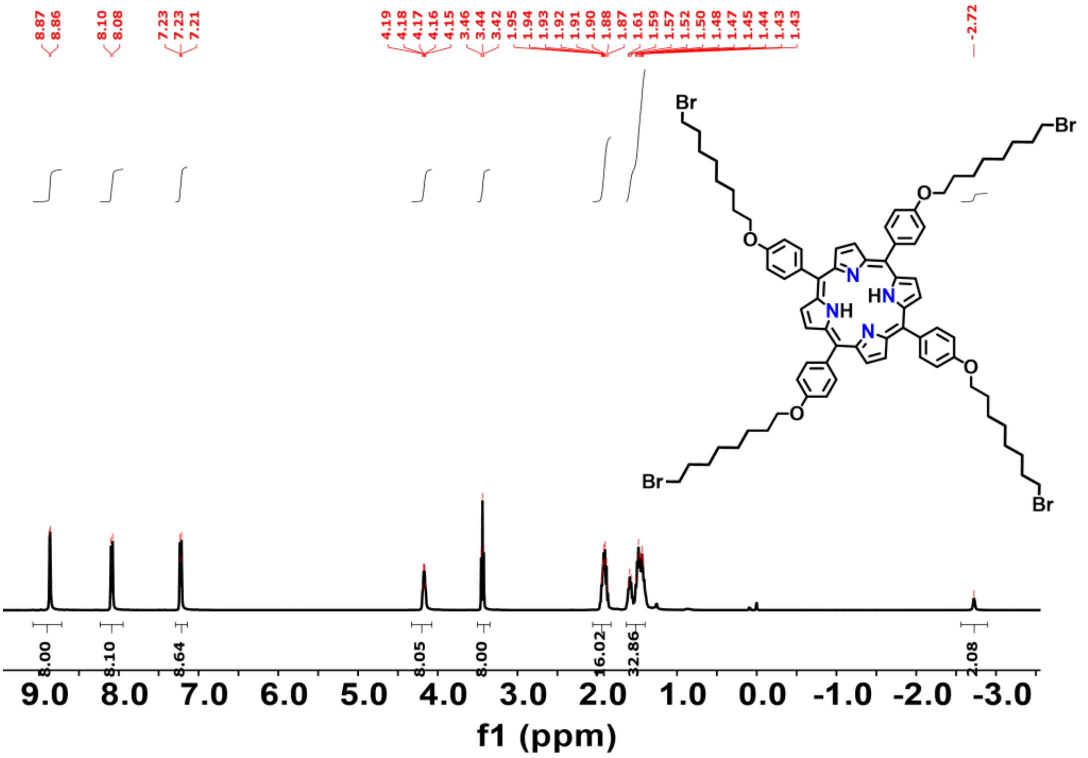


**Figure S29.** $^{1}H$ NMR spectra of **C8b** in $CDCl_3$.

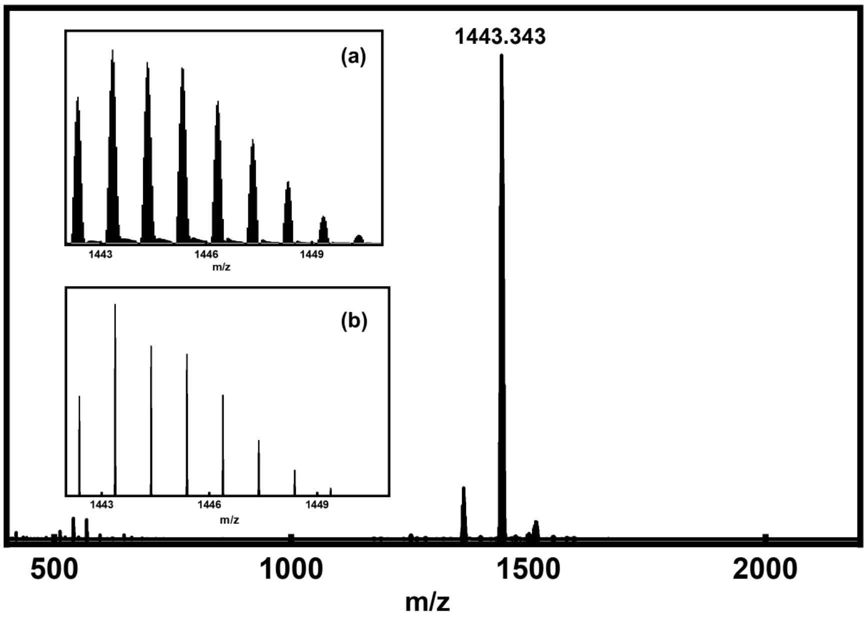


**Figure S30.** (a) Experimental and (b) simulated MALDI-TOF spectra of **C8b**. The inset shows an enlarged view of the mass spectrum.

xiii.**Synthesis of 2,2-(Dipicolylamino)-5,10,15,20-tetrakis-(4-(8-bromoctoxy)phenyl) porphyrin (C8DP)**: A mixture of 5,10,15,20-tetrakis(4-(8-bromooctoxy)phenyl)porphyrin (100 mg, 0.00693 mmol), $K_2CO_3$ (48.4 mg, 0.00035 mol), and 2,2′-dipicolylamine (0.075 mL, 0.4158 mmol) was dissolved in 3 mL of dry DMF. A solution of KI (58.1 mg, 0.00035 mmol) in 2 mL DMF was then added dropwise over 1 h to the porphyrin mixture maintained at 75 °C. The reaction was stirred for an additional 4 h at the same temperature. After completion, the mixture was diluted with 1 N HCl (15 mL) and washed twice with DCM. The aqueous phase was subsequently extracted using 4 N NaOH (50 mL) and twice with DCM. The combined organic layers were washed with water, dried over anhydrous sodium sulfate, and concentrated. Repeated washing of the crude product with hexane afforded the final product in 58% yield. The compound was characterised by 1H NMR spectroscopy.

$^1$H NMR (400 MHz, Chloroform-*d*) δ 8.79 (s, 8H), 8.46 (s, 8H), 8.04 (s, 8H), 7.30 (m, 16H), 7.18 – 6.92 (m, 16H), 4.8 (m, 8H), 4.5 (m, 8H), 4.1 (m, 8H), 2.89 (m, 8H), 1.89 (m, 16H), 1.68 (m, 16H), 1.42 (m, 16H) -2.82 (s, 2H).

$^{13}$C NMR (101 MHz, Chloroform-*d*) δ 160.22, 157.91, 156.04, 148.29, 148.03, 135.74, 134.57, 133.36, 122.16, 122.1, 121.10, 119.89, 118.79, 111.69, 67.19, 65.02, 63.07, 58.91, 53.55, 51.74, 51.30, 41.89, 28.65, 28.32, 27.95, 25.11, 13.10.

HRMS (ESI+): Calculated m/z for [C124H138N16O4] [M+H]+- 1917.1198 and found (M+H)+-1917.1175

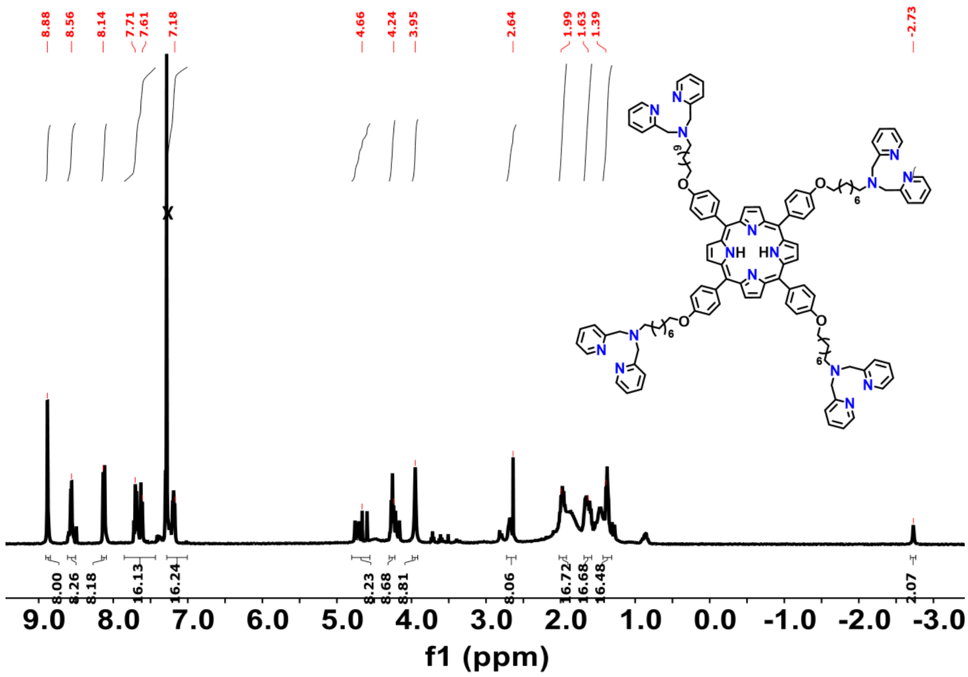

**Figure S31.** $^{1}$H NMR spectra of **C8DP** in $CDCl_3$. Peaks marked with “x” correspond to residual $CDCl_3$ (δ~ 7.26 ppm).

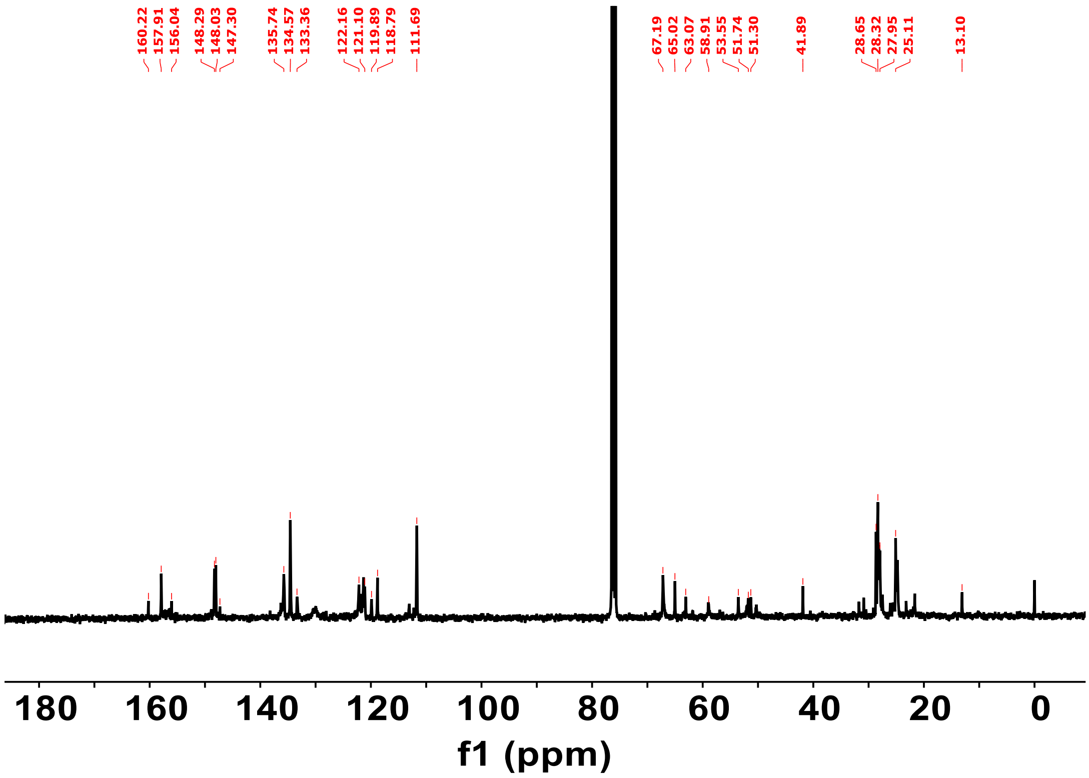

**Figure S32.** $^{13}$C NMR spectra of **C8DP** in $CDCl_3$.

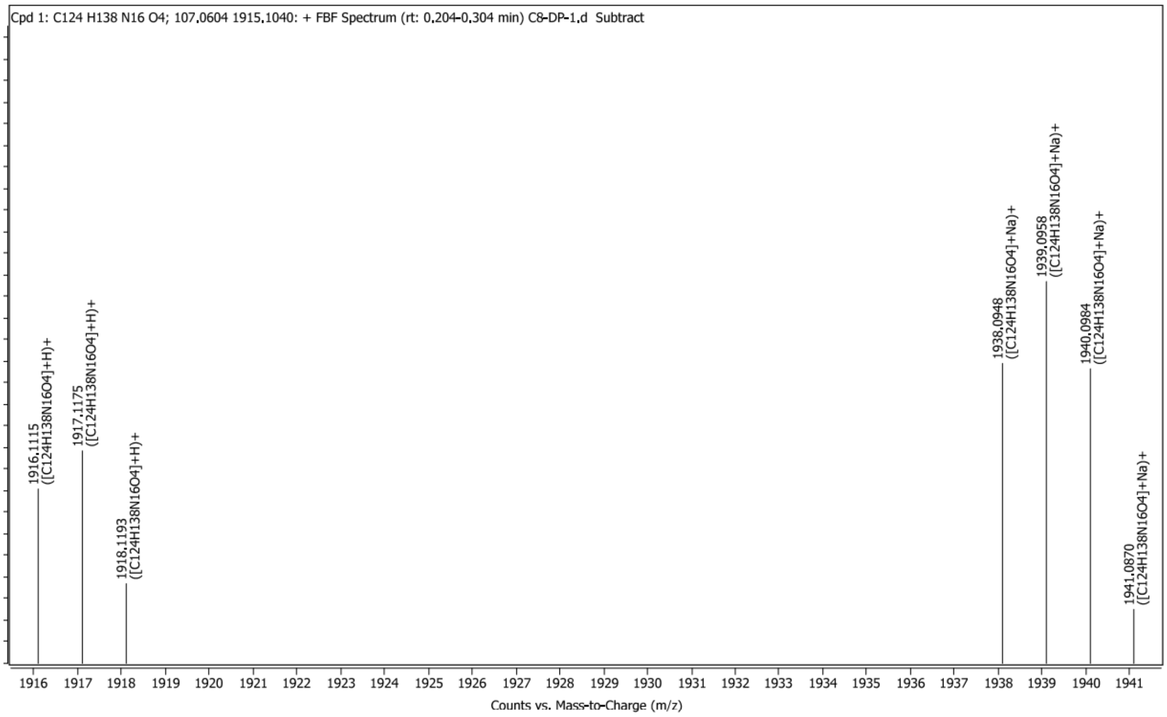


**Figure S33.** HRMS spectra of **C8DP**.

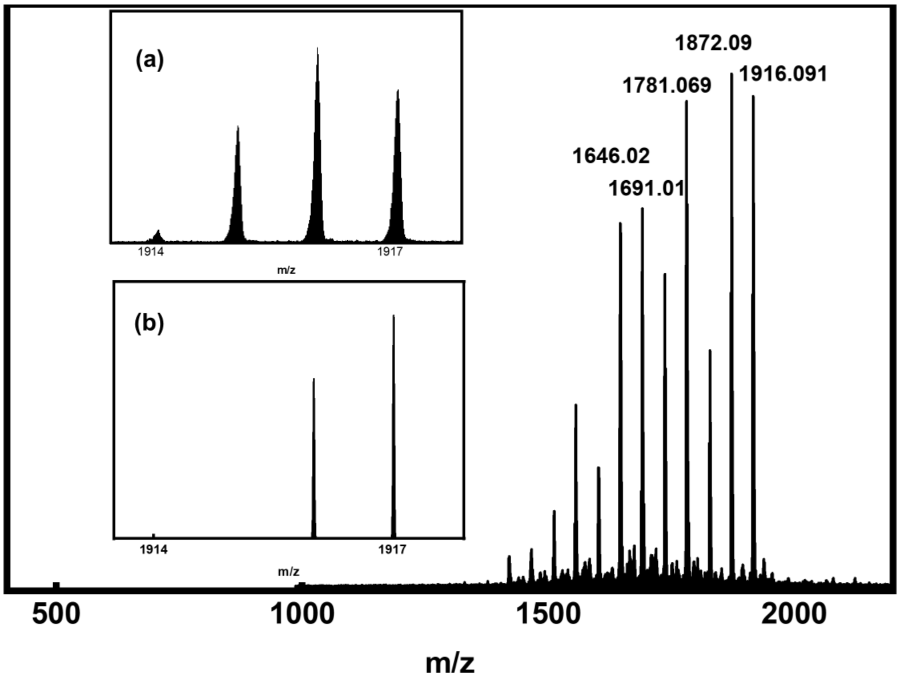


**Figure S34.** (a) Experimental and (b) simulated spectra of MALDI-TOF spectra of **C8DP**. The inset shows an enlarged view of the mass spectrum. Prominent fragment ions are observed at *m/z* 1781.069 and 1691.010, corresponding to the successive loss of the peripheral pyridylmethyl substituents ($\Delta m/z \approx 92$ for each fragmentation).

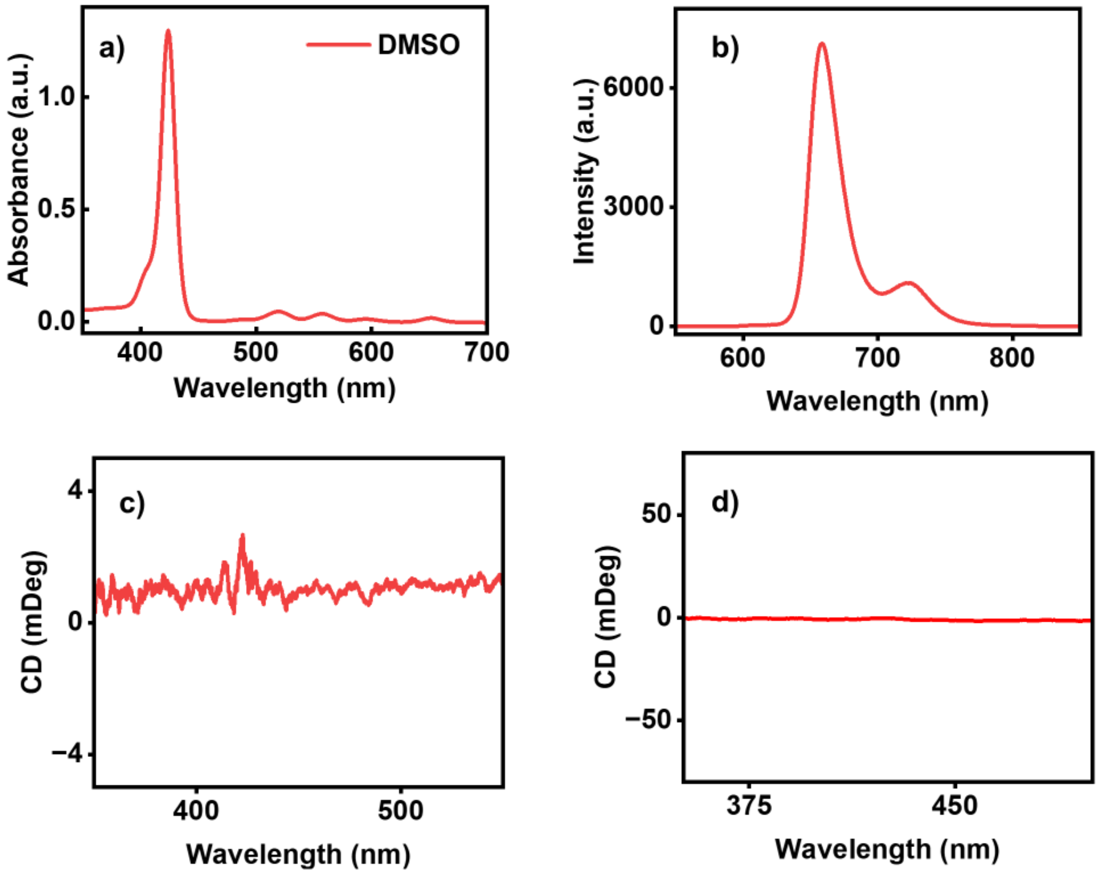


**Figure S35.** (a) UV-vis, (b) photoluminescence and (c) CD spectra of C3DP in DMSO. (d) CD spectra of **C3DP** in a mixture (1:99) DMSO:water.

Note: The CD plots in (c) and (d) correspond to the system in their monomeric and aggregated states, respectively.

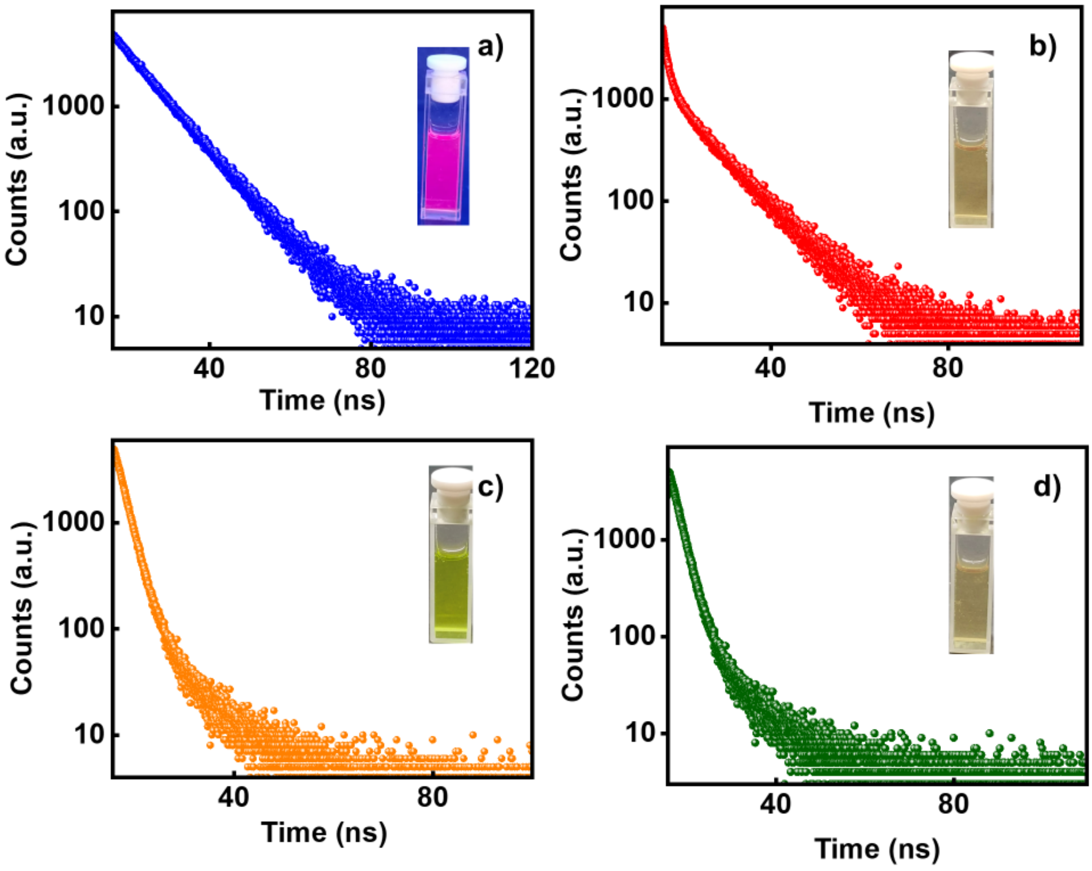


**Figure S36.** Fluorescence lifetime decay curves of C3DP in (a) DMSO. Fluorescence lifetime decay curves for (b) **C3DP**, (c) **C3DP-MA** and (d) **C3DP-MA-Zn** in DMSO:water (1:99) mixture.

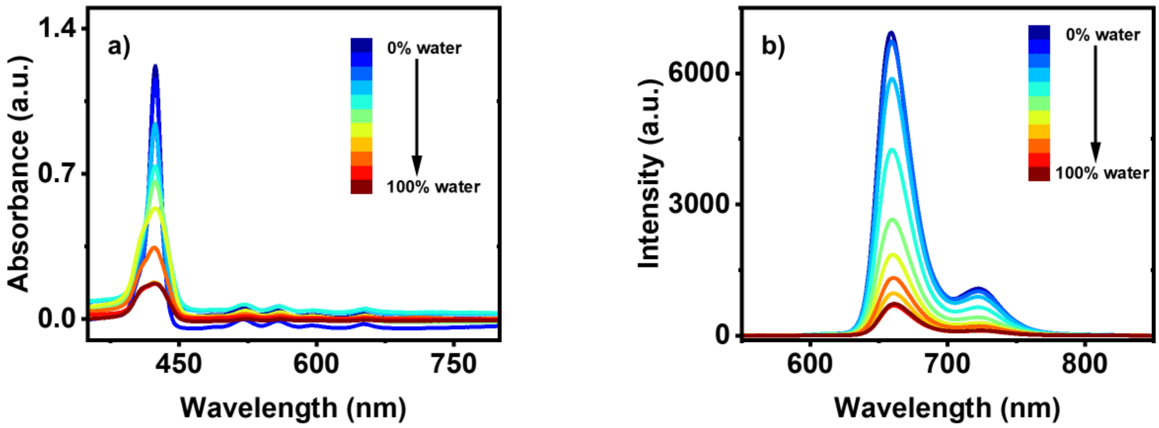


**Figure S37.** (a) UV-vis and (b) photoluminescence spectra of **C3DP** in varying composition of DMSO:water binary solvent mixture.

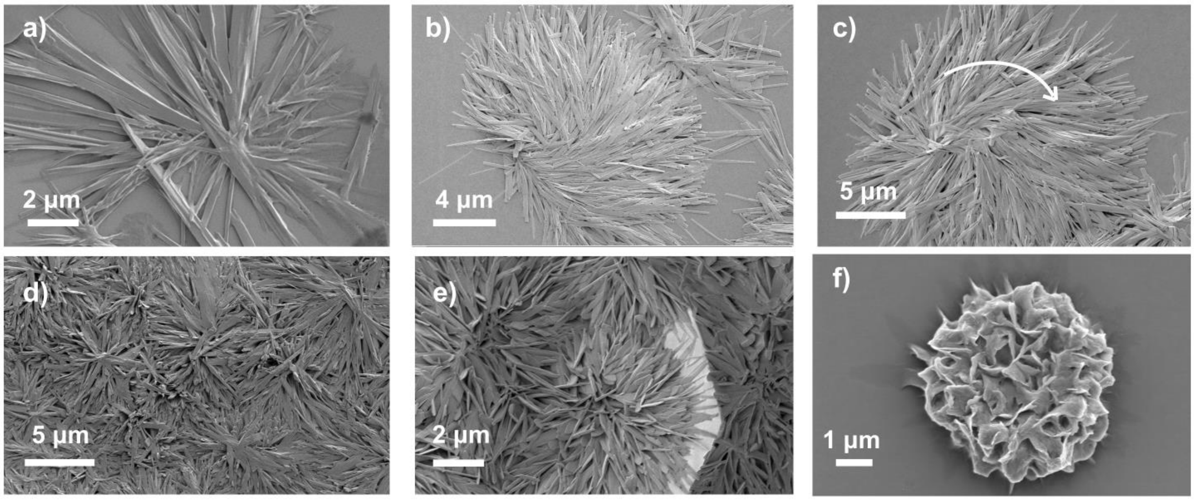


**Figure S38.** SEM images of **C3DP-DMA-Zn** assemblies on increasing the concentration of Zn(II); (a) 0 mM (b) 2.5 mM, (c) 5 mM, (d) 7 mM and (e) 12.5 mM and (f) 14.5 mM.

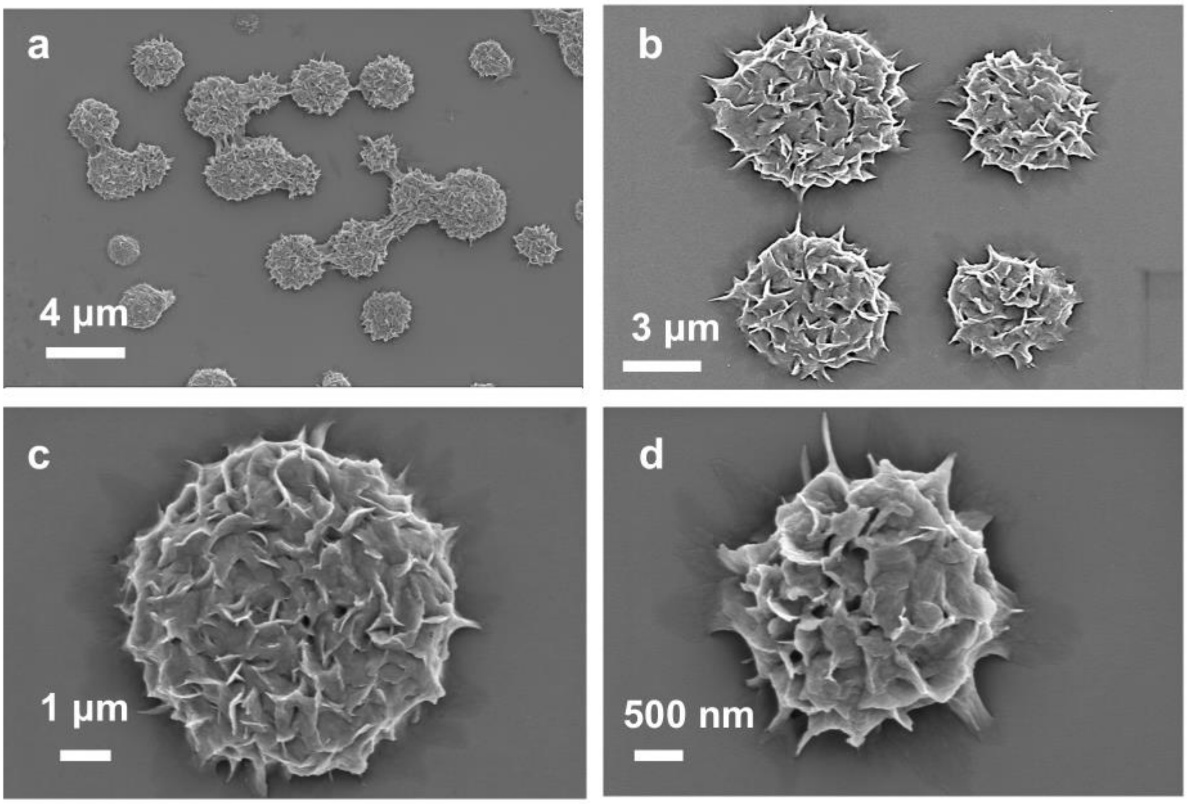


**Figure S39.** SEM images of **C3DP-DMA-Zn** in DMSO:water (1:99) mixture at different magnifications.

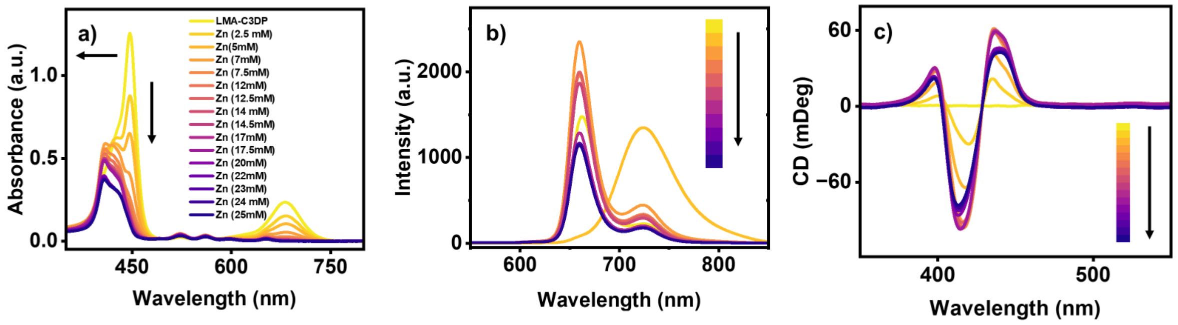


**Figure S40.** (a) UV-vis, (b) photoluminescence, and (c) CD spectral changes of **C3DP-LMA** assembly with increasing concentration of zinc.

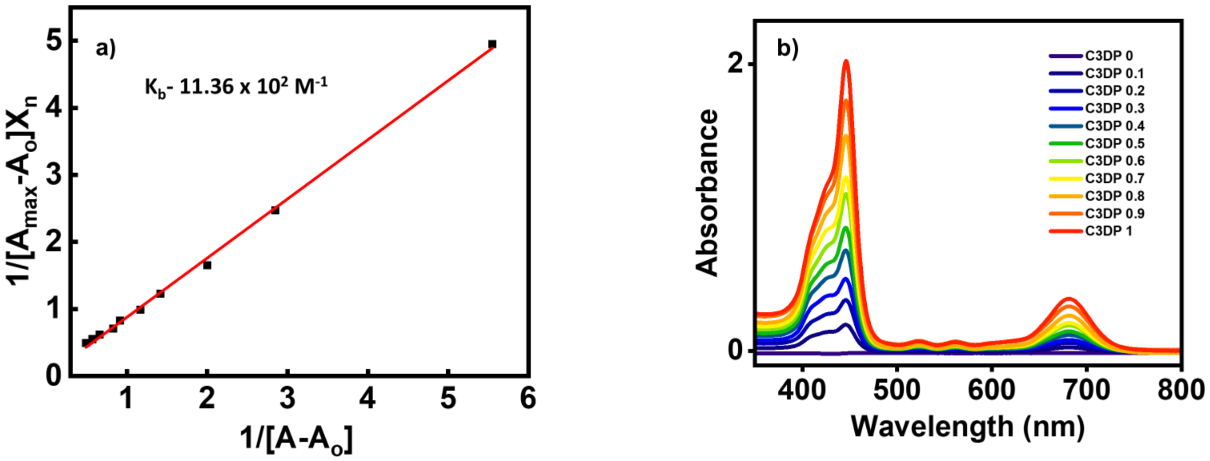


**Figure S41.** Benesi-Hilbrand plot for calculation of binding constant (Kb) and (b) the UV-Vis spectral changes on varying the **Zn:C3DP** ratio in water:DMSO (99:1).

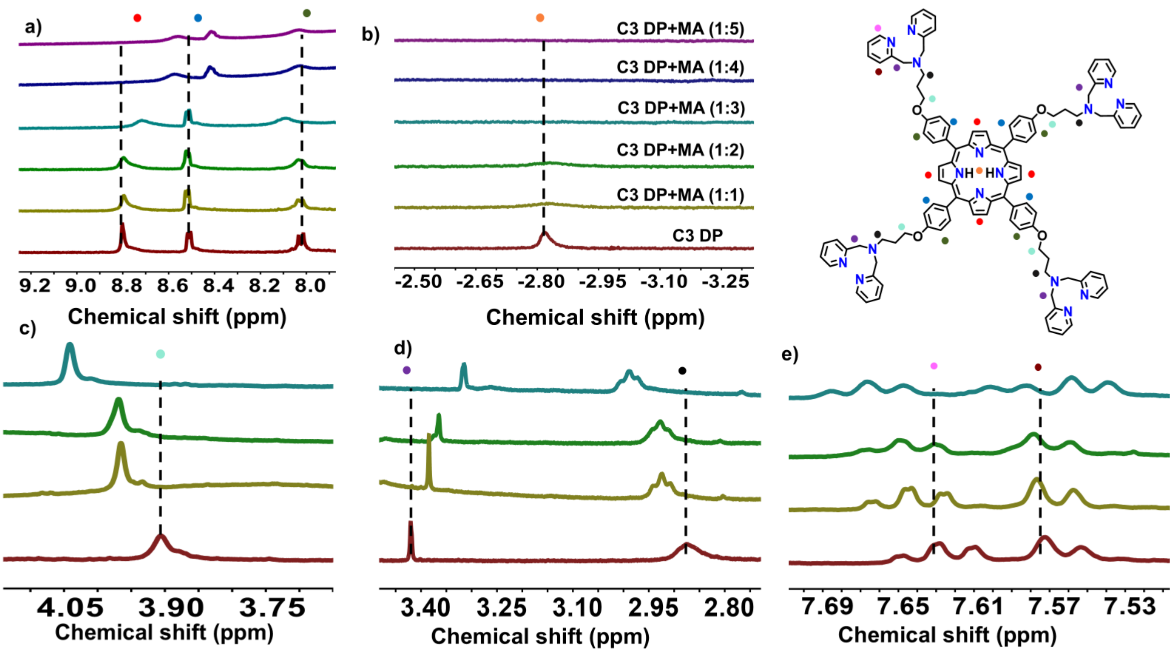


**Figure S42.** $^{1}$H NMR titration spectra of **C3DP** in DMSO-$d_6$ on gradual addition of MA in $D_2O$.

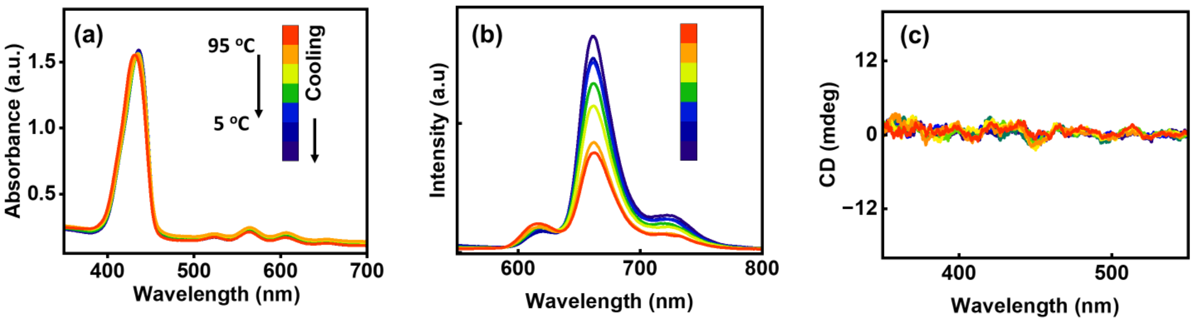


**Figure S43.** UV-vis, (b) fluorescence and (c) CD spectral changes on cooling down the heated sample of **C3DP-DMA-Z**n in DMSO:water (1:99) mixture from 95 °C back to 5 °C with each spectra monitored at an interval of 10 °C/min.

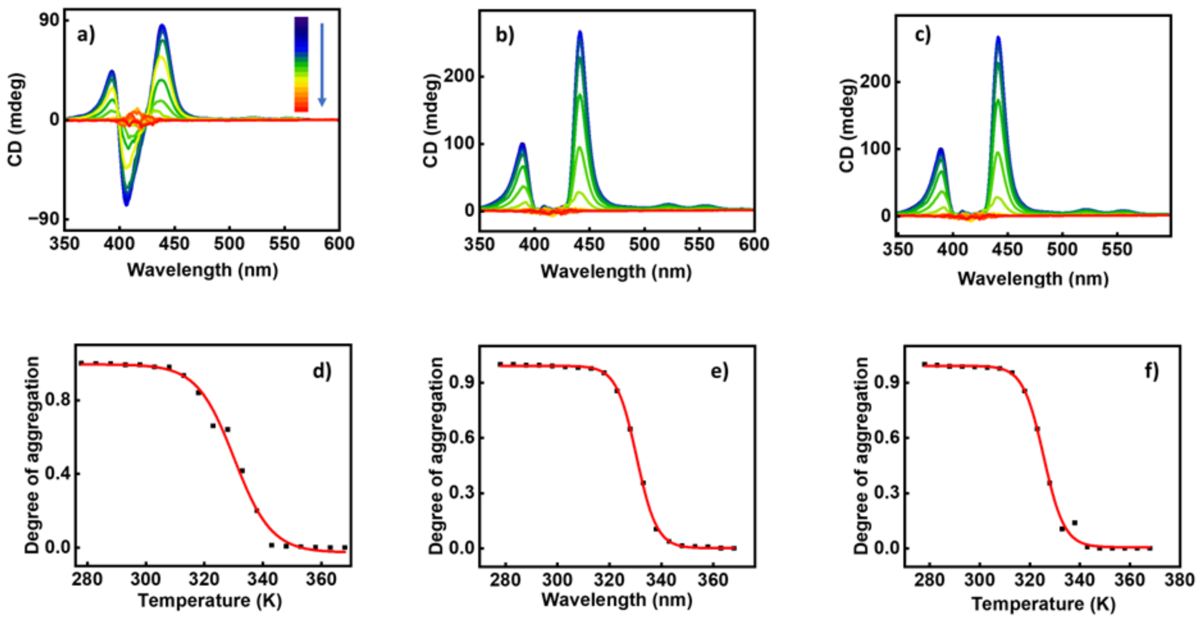


**Figure S44.** Temperature dependent UV-vis spectra and plot of degree of aggregation versus temperature of **C3DP-MA-Zn** aggregates at varying concentrations; (a,d) 16 µM, (b,e) 32 µM and (c,f) 48 µM, respectively in water.

**Table S1.** Table depicting the thermodynamic parameters obtained by fitting the degree of aggregation v/s temperature at varying concentrations.

| Conc. | ΔH | ΔS | Ka | ΔG | Tm |
|---|---|---|---|---|---|
| 5 µM | -216.0 KJ/mol | -532.7 J/mol | $2.09 \times 10^7$ $M^{-1}$ | -45 KJ/mol | 321.0 K |
| 16 µM | -233 KJ/mol | -595.0 J/mol | $1.6 \times 10^7$ $M^{-1}$ | -44 KJ/mol | 318.6 K |
| 32 µM | -230 KJ/mol | -580.0 J/mol | $9.6 \times 10^6$ $M^{-1}$ | -43 KJ/mol | 321.6 K |
| 48 µM | -232 KJ/mol | -585.0 J/mol | $3.6 \times 10^6$ $M^{-1}$ | -41 KJ/mol | 325.5 K |

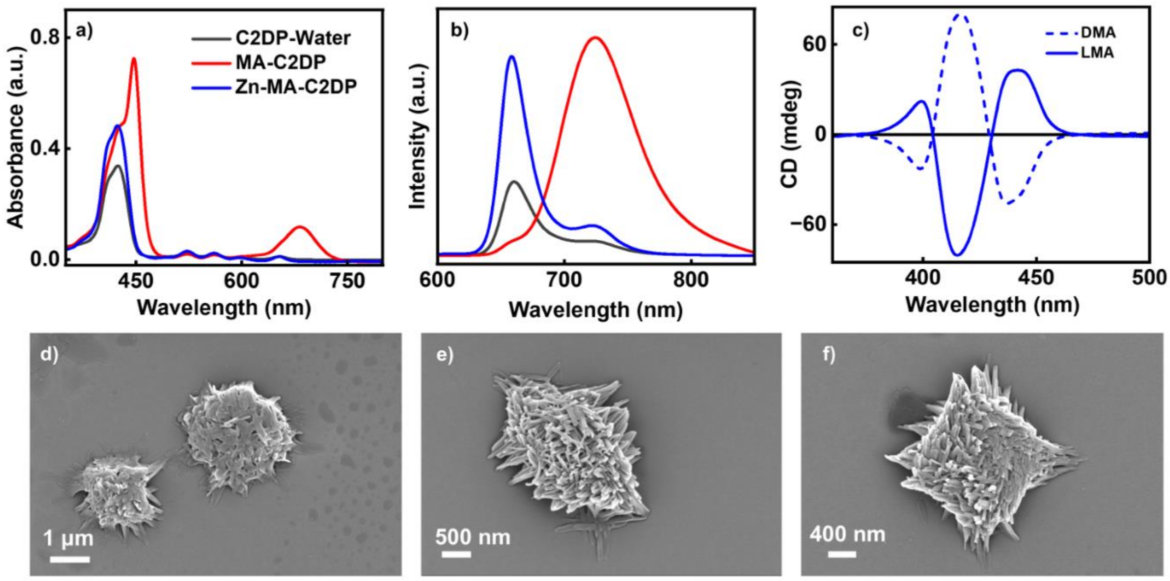


**Figure S45.** Uv-vis, (b) fluorescence and (c) CD spectra of C2DP in DMSO:water (1:99) on sequential addition of MA and zinc. (d-f) SEM images of **C2DP-MA-Zn** in DMSO:water (1:99) mixture.

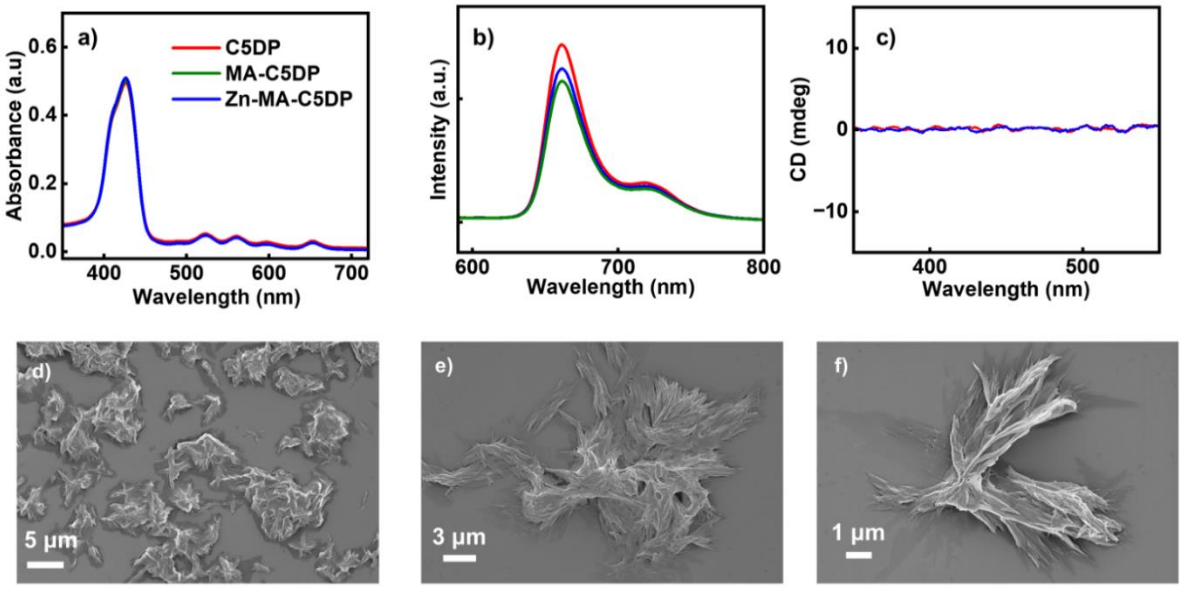


**Figure S46.** (a) UV-vis, (b) fluorescence and (c) CD spectra of C5DP in DMSO:water (1:99) mixture on sequential addition of MA and zinc. (d-f) SEM images of **C5DP-MA-Zn** in DMSO:water (1:99) mixture.

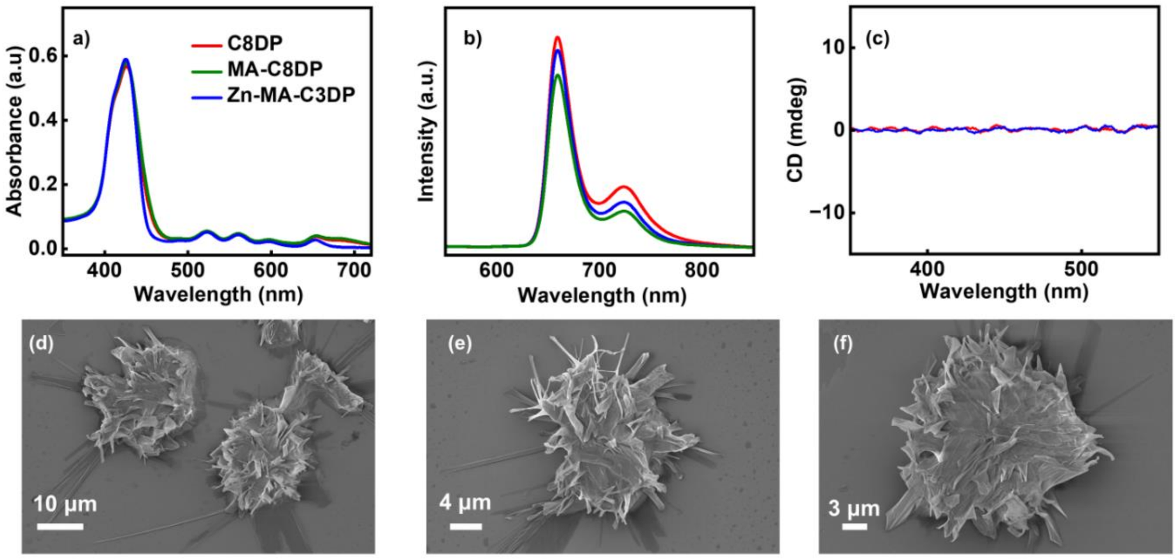


**Figure S47.** (a) UV-vis, (b) fluorescence and (c) CD spectra of C8DP in DMSO:water (1:99) mixture on sequential addition of MA and zinc. (d-f) SEM images of **C8DP-MA-Zn** in DMSO:water (1:99) mixture.

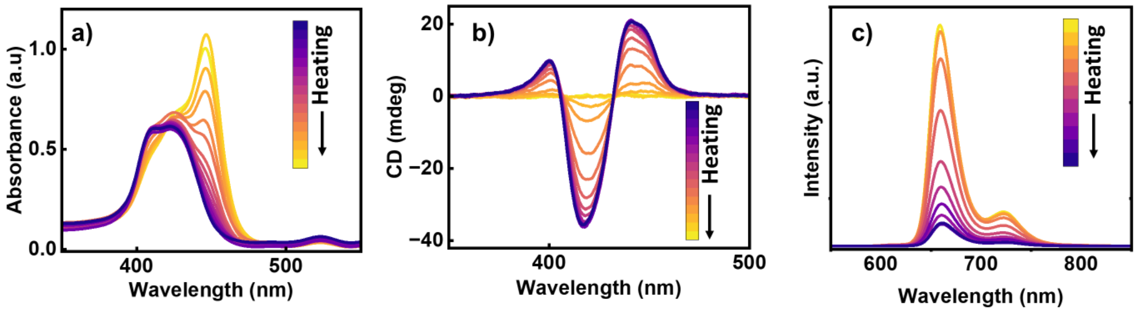


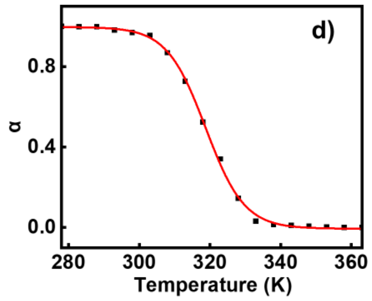


e)

| | ΔH | ΔS | Ka | ΔG | Tm |
|---|---|---|---|---|---|
| **C2DP-MA-Zn** | -170 KJ/mol | -438.76 J/mol | 7.58 x $10^6$ $M^{-1}$ | -39.248 kJ/mol | 318.605 |

**Figure S48.** Temperature dependent (a) UV-vis, (b) CD and (c) fluorescence spectra of **C2DP-MA-Zn** aggregates in water for sample heated from 5 °C to 95 °C with each spectra monitored at an interval of 10 ºC/min. (d) Plot of degree of aggregation versus temperature. (e) Table depicting the thermodynamic parameters obtained by fitting the degree of aggregation vs temperature.

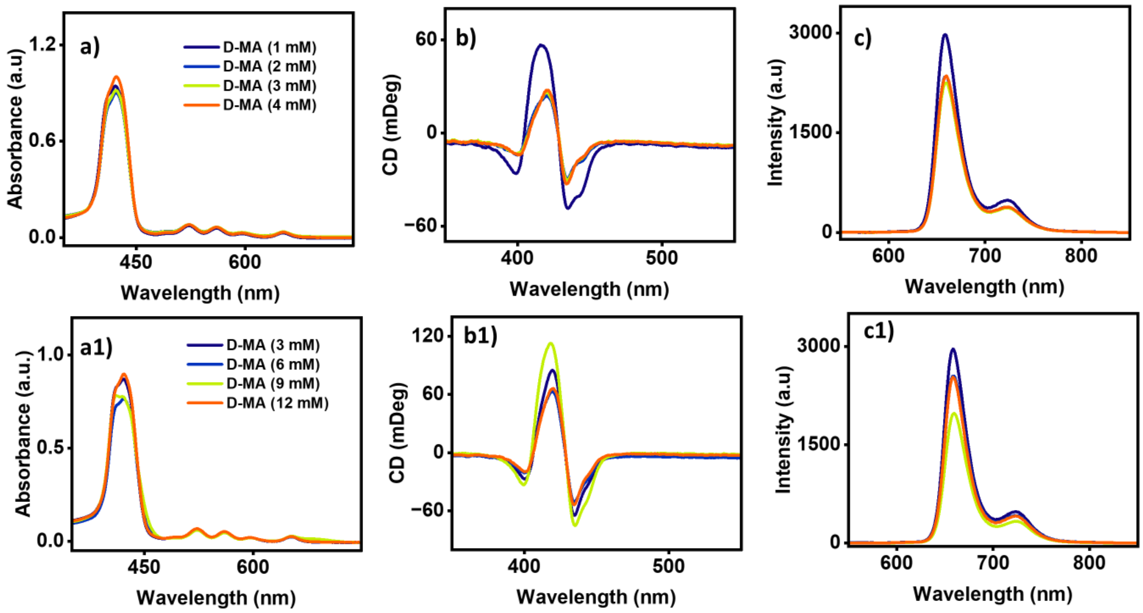


**Figure S49.** (a,a1) UV-vis, (b,b1) CD and (c,c1) Photoluminescence spectra of **C3DP-MA-Zn** with varying concentrations of D-MA.

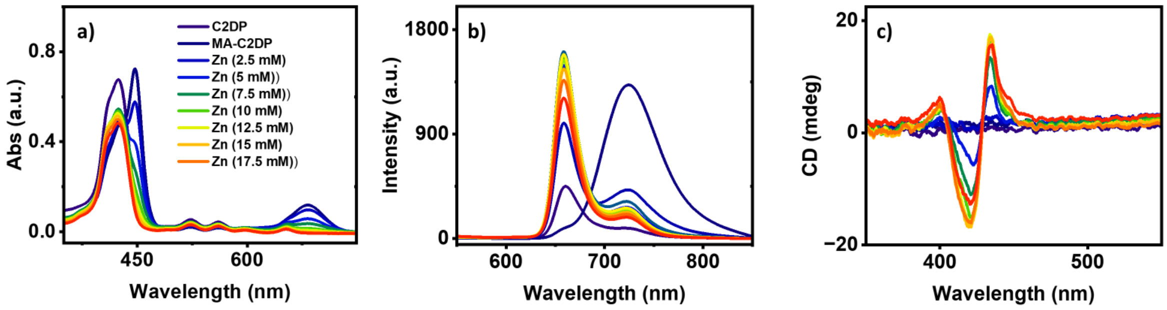


**Figure S50.** (a) UV-vis, (b) photoluminescence, and (c) CD spectral changes of **C2DP-LMA-Zn** assembly with increasing concentration of zinc.

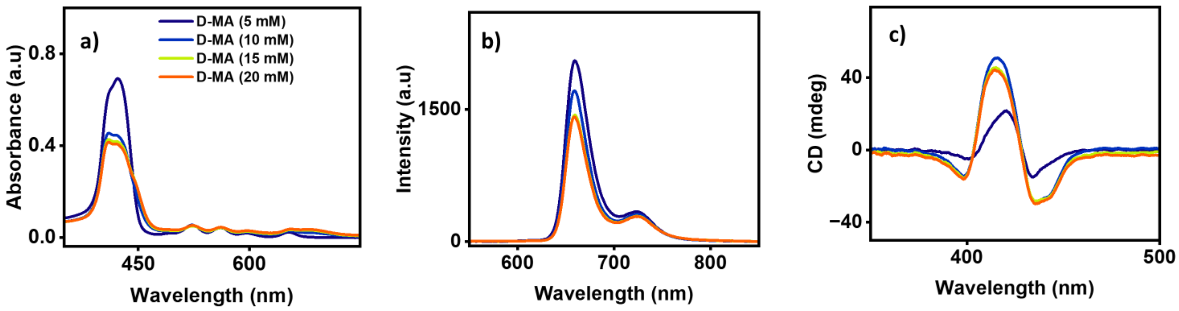


**Figure S51.** (a) UV-vis, (b) photoluminescence, and (c) CD spectra of **C2DP-DMA-Zn** with varying concentration of DMA.

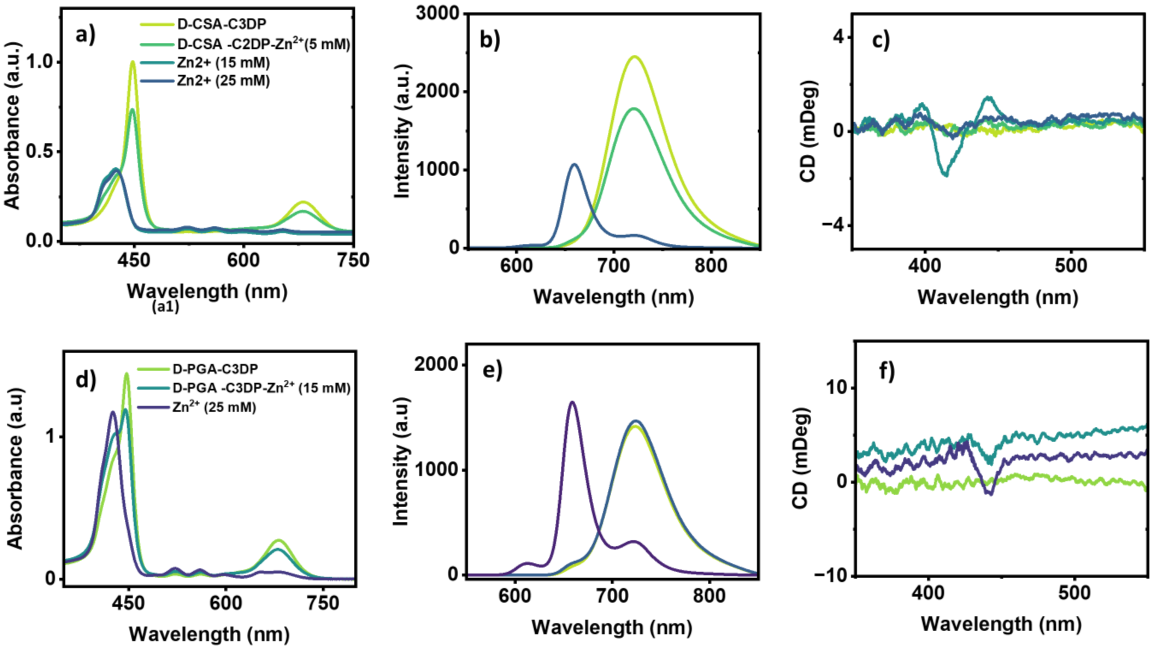


**Figure S52.** (a,d) UV-vis, (b,e) fluorescence and (c,f) CD spectra of **C3DP** with (a-c) camphor sulphonic acid and zinc , (d-f) polyglutamic acid and zinc.

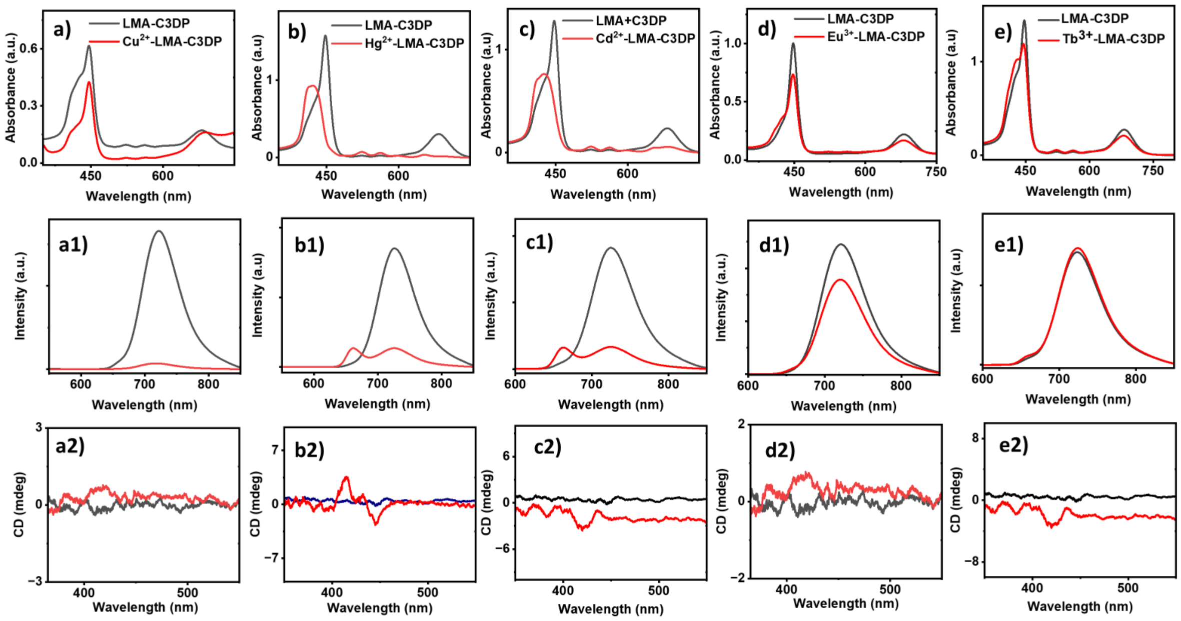


**Figure S53.** UV-vis, fluorescence and CD spectra of **C3DP-LMA** in the presence of (a-a2) $Cu^{2+}$, (b-b2) $Hg^{2+}$, (c-c2) $Cd^{2+}$, (d-d2) $Eu^{3+}$ and (e-e2) $Tb^{3+}$ respectively.

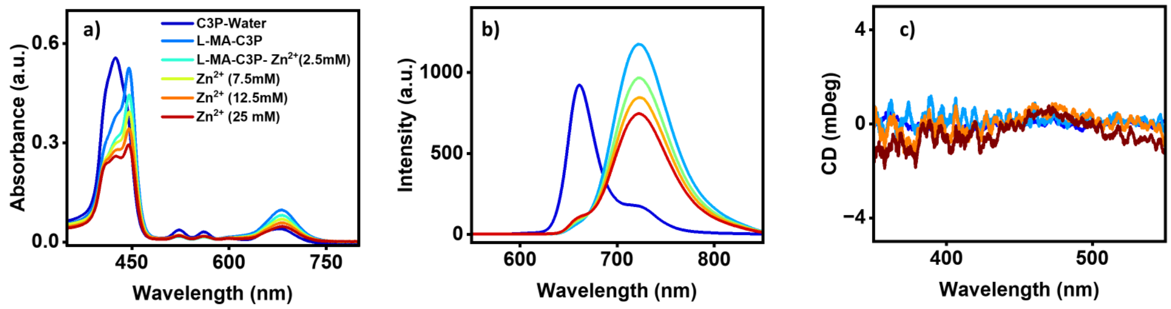


**Figure S54.** (a) UV-vis, (b) fluorescence and (c) CD spectra of **C3P** in water on gradual addition of MA and zinc.

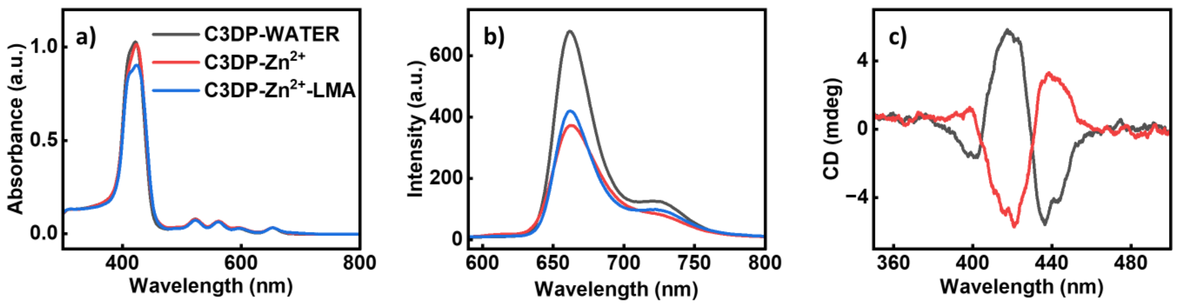


**Figure S55**. (a) UV-vis, (b) fluorescence and (c) CD spectra of **C3DP** in water on the initial addition of zinc followed by D-MA (black trace) and L-MA (red trace).

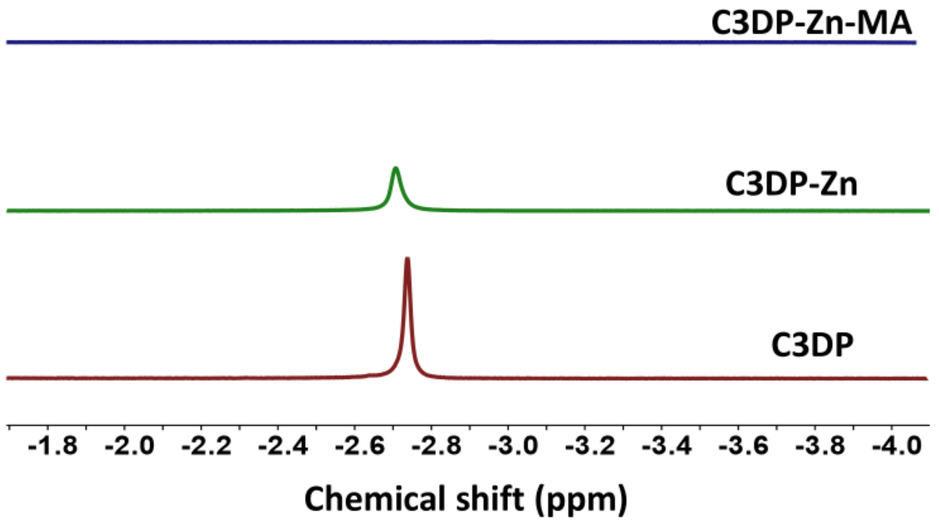


**Figure S56.** $^{1}H$ NMR titration spectra of **C3DP** in DMSO-d6 on gradual addition of zinc acetate followed by MA in $D_2O$.

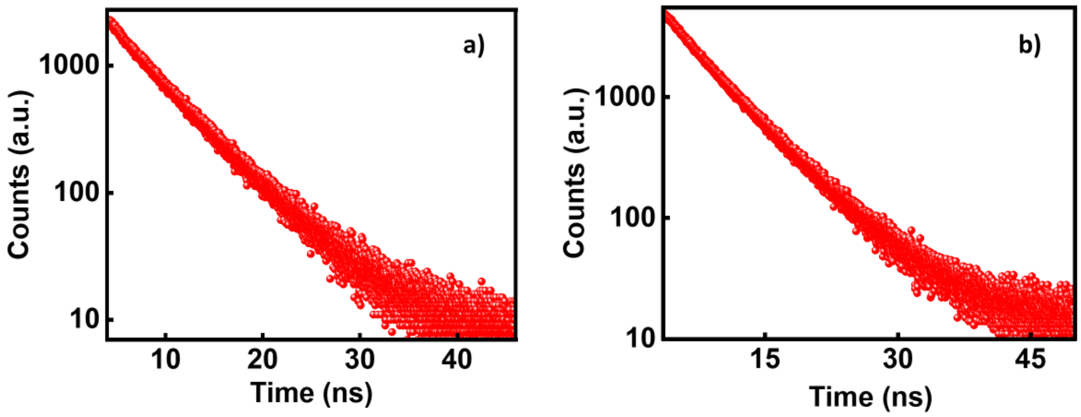


**Figure S57.** Fluorescence lifetime decay curves of PVA films incorporated with (a) **C2DP-MA-Zn** aggregates and (b) **C3DP-MA-Zn** aggregates.

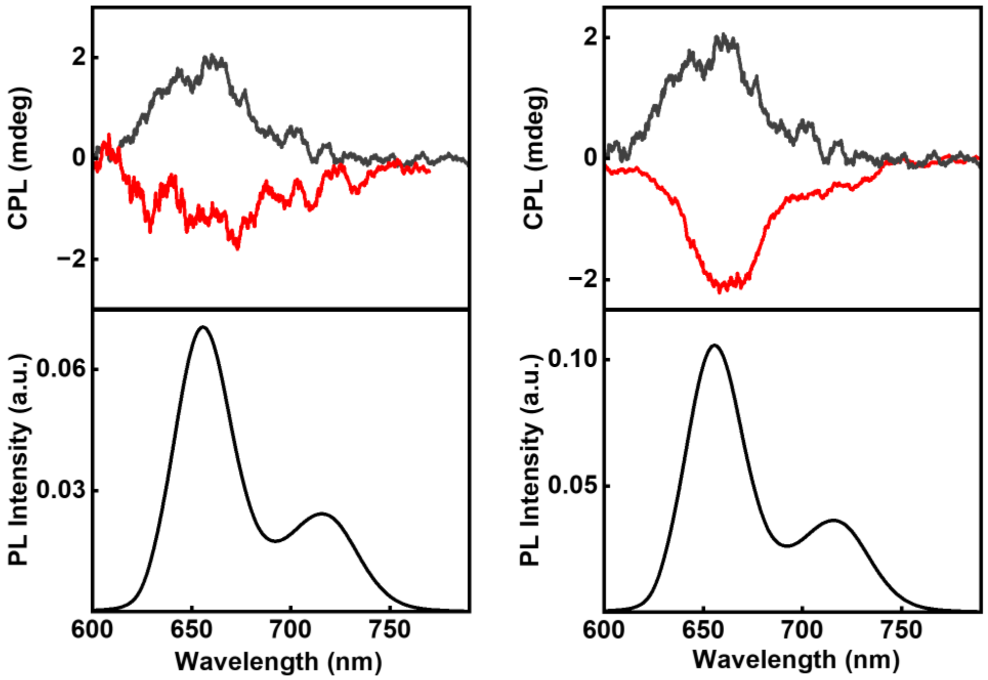


**Figure S58.** CPL spectra of **C2DP-MA-Zn** PVA film collected from multiple points.

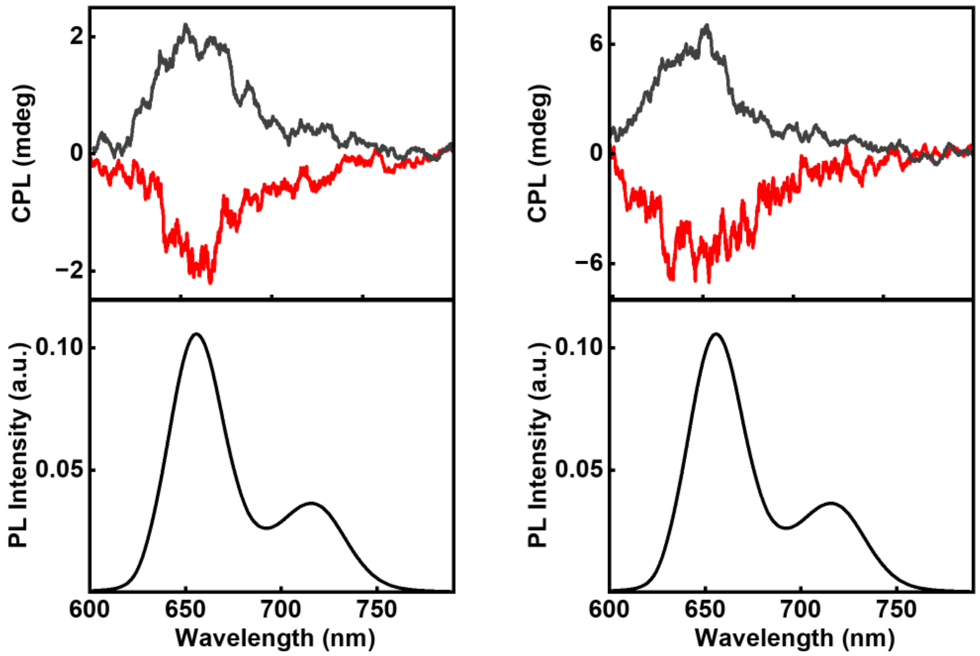


**Figure S59.** CPL spectra of **C3DP-MA-Zn** PVA film collected from multiple points.
